# Metabolome combined with gut microbiome revealed the lipid-lowering mechanism of Xuezhiping capsule on hyperlipidemic hamster induced by high fat diet

**DOI:** 10.3389/fmolb.2023.1147910

**Published:** 2023-02-20

**Authors:** Li Wang, Zhixin Zhang, Gan Luo, Ying Wang, Ke Du, Xiaoyan Gao

**Affiliations:** School of Chinese Materia Medica, Beijing University of Chinese Medicine, Beijing, China

**Keywords:** metabolome, microbiome, hyperlipidemia, liver, lipid metabolism, xuezhiping

## Abstract

**Introduction:** Hyperlipidemia is a common metabolic disorder with presence of excess fat or lipids in the blood, may induce liver injury, oxidative stress and inflammatory. Xuezhiping capsule (XZP) is a famous Chinese patent medicine clinically used for anti-hyperlipidemia. However, the regulation mechanism of XZP on hyperlipidemia has not been elucidated so far.

**Methods:** This study aimed to explore the effects of XZP on hypolipidemic, antioxidant and anti-inflammatory effects, and the potential mechanism by a combination of untargeted metabolomics and 16S rRNA sequencing.

**Results:** The results indicated that XZP reduced the level of total cholesterol (TC), triglyceride (TG), low density lipoprotein cholesterol (LDL-C), increased the level of high density liptein cholesterol (HDL-C), alleviated excessive accumulation of lipid droplets in liver. Biochemical indexes of liver function including gamma glutamyl transferase (GGT) and glutamic oxaloacetic transaminase (GOT) in liver were remarkably decreased. Meanwhile, XZP increased the level of oxidative stress biochemical indexes including superoxide dismutase (SOD) and glutathione (GSH). In addition, XZP increased the level of peroxisome proliferators-activated receptors α (PPARα), acetyl CoA carboxylase 1 (ACOX1) and cholesterol 7-alpha hydroxylase (CYP7A1) in liver, and improved lipid metabolism in serum, liver and fecal lipid metabolism. XZP increased diversity index and the ratio of Firmicutes and Bacteroidetes, regulated seventeen genera, and illustrated strong correlations with liver lipid metabolism and phenotypic indicators.

**Discussion:** These findings suggest that XZP reduced blood lipid and liver lipid, protected liver function, anti inflammation and anti-oxidation, ameliorate lipid metabolic disorders by modulating alpha linolenic acid and linoleic acid metabolism, bile acid metabolism, arachidonic acid metabolism, and regulated gut microbiota composition of high-fat diet (HFD) hamsters.

## 1 Introduction

Hyperlipidemia refers to a chronic disease caused by systemic metabolic disorder, characterized with abnormal plasma lipid levels. Hyperlipidemia induced by high-fat diet with lipid metabolism disorder including abnormal metabolism of bile acid, linolenic acid, and arachidonic acid (Bao et al., 2021; Xiao-Rong et al., 2021), changes of gut microbiota composition (Jia et al., 2021), and a systemic chronic low grade inflammation (Duan et al., 2018). XZP is a Chinese patent medicine composed of *Gynostemma pentaphyllum* (GP), *Crataegus pinnatifida* Bge (Hawthorn), *Fructus Rosa Roxburghii* (FRR), and *Cynanchum paniculatum*. These botanicals have abundant experimental studies on lowering blood lipid, antioxidant stress, antiinflammation, and regulating gut microbiota. However, there are relatively few studies on the above effects and mechanisms of XZP. The present study aimed to explore the effects and the potential mechanism of XZP on hyperlipidemic hamster by a combination of untargeted metabolomics and 16S rRNA sequencing.

The botanical drugs composed of XZP, including GP (Dai et al., 2022; Rao et al., 2022), Hawthorn (Peng et al., 2019; Feng et al., 2022) and FRR (Song & Shen, 2021), have been extensively studied in regulating TC, TG, LDL-C, and HDL-C. Researches showed that GP extract increased AMPK activation and suppressed adipogenesis by decreasing the mRNA expression of encoding CCAAT/enhancer binding protein-α (C/EBPα), peroxisome proliferator-activated receptors γ (PPARγ), sterol regulatory element-binding protein 1c (SREBP1C), and fatty acid synthase (FAS) (Park et al., 2014; Wang B. et al., 2020). In addition, GP extract elevated phosphatidylcholine metabolism decreased the level of trimethylamine N-oxide for antihyperlipidemia effect (Wang et al., 2013). Hawthorn (Shatoor et al., 2021) and vitexin (Peng et al., 2019) as a bioactive compound in Hawthorn leaf, both prevented high-fat diet-induced hepatic steatosis in rats by activating AMPK-induced suppression of SREBP1 and activation of PPARα. Research showed that FRR maintained metabolic homeostasis by regulating the metabolism of fatty acids, biosynthesis of BAs and steroids (Song & Shen, 2021). Hydroalcoholic extract of FRR improved the activities of lipoprotein lipase and hepatic lipase, downregulated the mRNA and protein expressions of SREBP1C and acetyl CoA carboxylase 1 (ACOX1), and upregulated the mRNA and protein expressions of PPARα in hepatic tissue (Wu et al., 2020; Ni et al., 2022). Thus, the present study tested TC, TG, LDL-C, and HDL-C in liver and serum, liver tissue oil red O staining, PPARα, ACOX1, and CYP7A1 in liver for evaluating the efficacy and potential mechanism of XZP in lipid-lowering.

High fat diet induced oxidative stress plays a crucial role in the development of hyperlipidemia (Tan & Norhaizan, 2019; Kasbi-Chadli et al., 2021). GP extract suppressed oxidative stress by increasing levels of sirtuin 6 and phase 2 anti-oxidant enzymes (Wang B. et al., 2020). The aqueous extract of Hawthorn reduced hepatic levels of reactive oxygen species, increased hepatic GSH and SOD levels (Hussain et al., 2021). Previous studies have shown that hydroalcoholic extract of FRR improved the activities of antioxidant enzymes (Wu et al., 2020) and increased colonic oxidative stress (Wang et al., 2022). The present study tested SOD, MDA and GSH in liver and serum for evaluating the anti-oxidative stress of XZP.

One of the consequences of excessive fat consumption contributes to not only a systemic low-grade inflammation but localized tissue dysfunction (Fatoorechi et al., 2016; Yu et al., 2019a; Malesza et al., 2021). GP extract ameliorated NASH by regulating gut microbiota and the TLR2/NLRP3 signaling pathway (Yue et al., 2022). Studies have shown that FRR repaired intestinal barrier dysfunction by reducing the levels of inflammatory cytokines, intestinal permeability in mice fed a high-fat diet (Wang et al., 2022). *Cynanchum paniculatum*, one of the botanicals in XZP, is commonly prescribed for the treatment of various inflammatory diseases by modulating of NF-κB and MAPK signaling pathway (Chen et al., 2020). The present study tested IL-6 and CRP in liver and serum for evaluating the antiinflammatory of XZP.

A high-fat diet induced to dysbiosis, gut barrier dysfunction, increased intestinal permeability, and leakage of toxic bacterial metabolites into the circulation (Malesza et al., 2021). Studies have shown that GP regulated short chain fatty acid metabolism and gut microbiota related to intestinal inflammation, and reduced the Firmicutes/Bacteroidetes ratio (Horne et al., 2020; Li S. et al., 2022). GP extract enriched the abundance of beneficial bacteria such as Lactococcus spp. and inhibiting the abundance of pathogenic bacteria such as Ruminococcus spp. in the gut (Shen et al., 2020). FRR decreased the ratio of Firmicutes to Bacteroidetes, and increased the abundance of Prevotella and Ruminococcus on hyperlipidemic rats (Ji et al., 2022), significantly affected bile acid, amino acid and lipid metabolism (Wang L. et al., 2020).

In this study, we analyzed the efficacy of XZP in reducing blood lipid and liver, protecting liver function, anti inflammation, and anti-oxidation in high fat diet hamsters. In addition, we focused on the analysis of XZP on lipid metabolism in serum, liver, and feces, and gut microbiota composition of hamsters induced by HFD. Finally, we analyzed the correlation between intestinal genera and liver lipid metabolism, and the correlation between genera phenotypic indexes of liver. The purpose of our research was study the regulation mechanism of XZP on hyperlipidemia by integrating phenotypic indicators, lipid metabonomics and gut microbiota.

## 2 Materials and methods

### 2.1 Experimental design and animal care

Eighty-five 6-week old 100 ± 10 g, male golden hamsters, purchased from Beijing Vital River Laboratory Animal Technology Co., Ltd (SCXK (Beijing) 2016-0011, NO.110011200109811516). The animal care procedures and testing were performed in accordance with the guidelines prepared by the Beijing University of Chinese Medicine Animal Center. Every three hamsters were housed in one cage with a comfortable environment of 25°C ± 1°C, 50%–60% humidity, and 12 h/12 h dark/light cycle. We analyzed the quality control analysis of XZP before the experiments (Supplementary Figure S1 **).**

Thirteen hamsters were fed with standard diet. At the same time, seventy-two hamsters were fed with high fat diet induced into hyperlipidemia models. Three weeks later, blood was collected from the orbit to test TC and TG. Three hamsters with higher TC and TG in the standard diet group were excluded, and ten hamsters were assigned into the normal control (NC). Fifty hamsters with significantly elevated TC and TG in serum than NC group were randomly split into five groups of ten hamsters each, HFD (treated with water, 1.0 mg/kg/day), Atorvastatin group (Pfizer Inc., NO. DP6613, 2.5 mg/kg/day), low-dose XZP (Guizhou Taihe Pharmaceutical Co., Ltd, NO.20200801-2, XZP-L, 0.225 mg/kg/day), middle-dose XZP (XZP-M, 0.45 mg/kg/day) and high-dose XZP group (XZP-H, 0.9 mg/kg/day). Hamsters of ATVTT and XZP groups were treated at 8:30 every morning for 4 weeks, while hamsters in NC and HFD groups were treated with same water. During the treatment, NC group fed with standard diet, HFD and treatment groups fed with high fat diet. High fat diet was provided by Jiangsu Xietong Pharmaceutical Bio-engineering Co., Ltd (NO. 20201026). It contained 41% standard diet, 20% fructose, 18% lard oil, 15% casein, 2% dicalcium phosphate, 2% mineral mix, 0.5% sodium cholate and 1.5% cholesterol. High fat diet was used for this study containing 41% of total calories (Kcal %) from fat, 20% from proteins, and 39% from carbohydrates.

### 2.2 Sample collection and preparation

The body weights and food intake were measured weekly. All the hamsters were survived during the duration of the experiment. After 7 weeks, individual hamsters were placed in metabolic cages (1 per cage) to obtain 24-h fecal collections, and fecal samples were stored at −80°C before analysis. At the end of the experiment, hamsters were anesthetized using sodium pentobarbital by intraperitoneal injection at the dosage of 30 mg/kg body weight. Blood samples were was collected from hepatic portal vein of hamsters with blood collection vessel without anticoagulant. The liver samples were carefully excised and weighed, immediately snap frozen using liquid nitrogen, and then stored at −80°C for metabolomic analysis. A viscera index was calculated using the formula: organ weight/body weight (mg/g).

### 2.3 Biochemical assay of blood and liver

TC (Solarbio, NO. 20201201), TG (Solarbio, NO. 20201120), HDL-C (mlbio, NO.11/2020 04/2021), and LDL-C (mlbio, NO. 04/2021) levels in the blood and liver samples were measured to evaluate the expression of lipid substances. GPT (Solarbio, NO. 20201125), GOT (Solarbio, NO.20201130), GGT (mlbio, NO.04/2021) and AKP/ALP (Solarbio, NO.20201201) levels in the blood and liver samples were measured to evaluate liver function. SOD (Solarbio, NO. 20201123) and MDA (Solarbio, NO.20201111) levels in the blood and liver samples were measured to evaluate the ability of the hamsters to resist oxidative stress. GSH (Solarbio, NO.20201110), IL-6 (mlbio, NO.11/2020) and CRP (mlbio, NO.11/2020) levels in the blood samples were measured to evaluate the anti-inflammatory function of the hamsters.

### 2.4 Histological examination of liver

After the frozen sections of liver were fixed, oil red O staining, background differentiation, hematoxylin staining and sealing were performed in turn. Lipid deposition in liver was observed by oil red O staining. HE staining examined the degree of liver tissue damage. Use CaseViewer 2.2 scanning software to select the liver area for 200x imaging. During imaging, try to fill the entire field of vision with tissues to ensure that the background light of each photograph is consistent. After imaging, use Image Pro Plus 6.0 analysis software to measure the pixel area of oil red grease drops in each picture and the corresponding pipe wall pixel area and calculate the proportion of oil red grease drops in a unified unit pixel.

### 2.5 Western blot

Liver was crushed by a Refrigerated High-Speed Homogenate Machine with RIPA buffer on ice for 60 s and then centrifuged at 13,000 xg at 4°C for 30 min. The protein concentration of the supernatant of liver homogenate was detected by BSA assay. Equal amounts of protein were resolved by SDS-Polyacrylamide gel electrophoresis and transferred onto Immun-Blot PVDF Membrane. Then, 5% NFDM in TBST as blocking buffer blocked the membrane at room temperature for 1 h. The membrane was cut and each part was incubated with corresponding antibody (PPARα, NO. ab8224, diluted with TBST solution at 1:1000; ACOX1, proteintech, NO.15540-1-AP, diluted with TBST solution at 1:4000; CYP7A1, BOSTER, NO. A01601, diluted with TBST solution at 1:500) at room temperature for 1 h and at 4°C overnight in quick succession. Then, membrane cleaned with TBST 4 times. The membrane was further incubated with corresponding secondary antibodies (Goat Anti-Mouse IgG H&L (HRP), 1:20000, abcam, NO. ab205719) at room temperature for 1 h. Blots were incubated with the primary antibody followed by horseradish peroxidase-conjugated secondary antibody. Results were detected by FluorChem E Imaging System (proteinsimple, United States of America). The protein expression levels of PPARα, ACOX1 and CYP7A1 were normalized with *ß*-actin and quantified.

### 2.6 UPLC–MS analysis of metabolites from the feces, liver and serum

In order to precipitate protein and extract metabolites, 100 μL serum samples of each experimental group added of 400 μL methanol: acetonitrile (volumetric ratio of 1:1) to vortex 30 s, stored at 4°C for 2 h. The homogenized samples were then centrifuged for 20 min at 13500 xg, after which the supernatant was transferred to EP tubes and blow dry with nitrogen blowing instrument. Take 20 μL of each group of samples and mix them into QC serum samples, and use the same preparation method to prepare QC samples. Finally, the residue was reconstituted with 200 μL methanol: acetonitrile (1:1), centrifuged for 20 min at 13500 xg, centrifuged for 20 min at 13500 xg, and an aliquot of 3 μL was injected for UPLC–Q-TOF/MS analysis.

Liver sample weighing 80 mg was transferred to 2 mL grinding tube containing 50 mg Zirconia beads. Liver homogenate were obtained by addition of 200 mL of ultra pure water to grinding tube and homogenized for 30 s in low temperature grinder at maximum speed. The homogenized samples added of 400 μL methanol: acetonitrile (volumetric ratio of 1:1) to vortex 30 s, then extract for 30 min by ultrasonic extractor at low temperature. The homogenized samples were then centrifuged for 20 min at 13500 xg, after which the supernatant was transferred to EP tubes and blow dry with nitrogen blowing instrument. Take 20 mg of each group of samples and mix them into QC liver samples, and use the same preparation method to prepare QC samples. Finally, the residue was reconstituted with 200 μL methanol: acetonitrile (1:1), centrifuged for 20 min at 13500 xg, and an aliquot of 3 μL was injected for UPLC–Q-TOF/MS analysis.

Fecal sample weighing 80 mg was added into 2 mL grinding tube containing 50 mg Zirconia beads. Fecal homogenate were obtained by addition of 200 mL of ultra pure water to grinding tube and homogenized for 30 s in low temperature grinder (Servicebio KZ-III-F) at maximum speed. The homogenized samples added of 400 μL methanol: acetonitrile (volumetric ratio of 1:1) to vortex 30 s, then extract for 30 min by ultrasonic extractor (KQ-600DE, Kunshan Ultrasonic Instruments Co. Ltd., Kunshan, China) at low temperature. The homogenized samples were then centrifuged for 20 min at 13500 xg, after which the supernatant was transferred to EP tubes and blow dry with nitrogen blowing instrument. Take 40 mg of each group of samples and mix them into QC fecal samples, and use the same preparation method to prepare QC samples. Finally, the residue was reconstituted with 200 μL methanol: acetonitrile (1:1), centrifuged for 20 min at 13500 xg, and an aliquot of 3 μL was injected for UPLC–Q-TOF/MS (Thermo Q Exactive Orbitrap) analysis.

Samples were analysed using an Thermo Scientific Ultimate 3,000 system coupled to an Thermo Scientific QExactive plus with Waters BEH C18 (1.7 μm, 100 mm) using the same method described above. Eluent A consisted of 0.1% formic acid (v/v) in water and eluent B consisted of 0.1% formic acid (v/v) in 100% acetonitrile. The analytical gradient was: 0 min, 1% A; 2 min, 35% A; 7 min, 75%A; 10 min, 99% A; 11 min, 99% A; 16 min, 1% A. Flow rate was 0.4 mL/min with an injection volume of 3 μL. Samples were held at 4°C in the autosampler, and the column was operated at 45°C. The MS operated in positive ionization mode and negative ionization mode with capillary voltage set to 3.5 KV and 2.8 KV. Capillary temperature and Aux gas heater temperature in positive and negative ion mode were both set to 320°C. Scan range was150–1200 m/z.

### 2.7 16S rRNA profiling of the gut microbiota

Bacterial RNA from feces of ten hamsters from each group was extracted using CTAB/SDS method. RNA concentration and purity was monitored on 1% agarosegels. According to the concentration, RNA was diluted to 1 ng/μL using sterile water. For each sample, 16S rRNA genes were amplified with the specific primer with the barcode. Primer contained 16S V3-V4 (341F-806R), 18S V9 (1380F-1510R) and ITS1(ITS1F- ITS2R). All PCR reactions were carried out in 30 μL reactions with 15 μL of Phusion ® High-Fidelity PCR Master Mix (New England Biolabs); 0.2 μM of forward and reverse primers, and about 10 ng template RNA. Thermal cycling consisted of initial denaturation at 98°C for 1 min, followed by 30 cycles of denaturation at 98°C for 10 s, annealing at 50 °C for 30 s, and elongation at 72°C for 60 s. Finally 72°C for 5 min. Mix same volume of 1X loading buffer (contained SYB green) with PCR products and operate electrophoresis on 2% agarose gel for detection. Samples with bright main strip between 400 and 450bp were chosen for further experiments. PCR products mixed in equidensity ratios and purified with AxyPrep RNA Gel Extraction Kit (AXYGEN). Sequencing libraries were generated using NEB Next® Ultra™ RNA Library Prep Kit for Illumina (NEB, United States of America) following manufacturer’s recommendations and index codes were added. The library quality was assessed on the Qubit@ 2.0 Fluorometer (Thermo Scientific) and Agilent Bioanalyzer 2100 system. At last, the library was sequenced on an Illumina Miseq/HiSeq2500 platform and 250bp/300bp paired-end reads were generated.

### 2.8 Statistical analysis

Raw data of experiment processed by Progenesis QI v2.0 (Non-linear Dynamics, Newcastle, U.K.) for visualization, processing, and interpretation of multidimensional LC–MS data. LC-MS data imported to Progenesis QI for peak picking and alignment acquired, and created principal components analysis (PCA) and orthogonal partial least square-discriminant analysis (OPLS-DA) and further confirmed using analysis of ANOVA. Metabolite peaks were assigned by MS/MS analysis combined with the Mass Fragment TM application manager (Waters corp., Milford, United States). Available biochemical databases including the Human Metabolome Database (HMDB) (http://www.hmdb.ca/) and KEGG (http://www.genome.jp/kegg/) used for bioinformatics analysis. Further multivariate statistical analysis was conducted using the SIMCA software (Version 14.1, MKS Data Analytics Solutions, Umea, Sweden). Orthogonal projections to latent structures-discriminate analysis (OPLS-DA) was applied to visualize group differences and obtain variables responsible for group separation.

The data analysis of 16S rRNA requires paired-end reads assemblies. Paired-end reads from the original RNA fragments were merged using FLASH with a very fast and accurate analysis tool, which was designed to merge paired-end reads when at least some of the reads overlap the read generated from the opposite end of the same RNA fragment. OTU clustering and species annotation was performed. Sequences analysis was performed by UPARSE software package using the UPARSE-OTU and UPARSE-OTU ref algorithms. In-house Perl scripts were used to analyze alpha (within samples) and beta (among samples) diversity. Sequences with ≥97% similarity were assigned to the same OTUs. We pick a representative sequences for each OTU and use the RDP classifier to annotate taxonomic information for each representative sequence. In order to compute Alpha Diversity, we rarify the OTU table and calculate three metrics: Chao1 estimates the species abundance; Observed Species estimates the amount of unique OTUs found in each sample, and Shannon index. Rarefaction curves were generated based on these three metrics. The third is to analyze the phylogenics distance and community distribution. Graphical representation of the relative abundance of bacterial diversity from phylum to species can be visualized using Krona chart. Cluster analysis was preceded by principal component analysis (PCA), which was applied to reduce the dimension of the original variables using the QIIME software package. QIIME calculates both weighted and unweighted unifrac distance, which are phylogenetic measures of beta diversity. We used unweighted unifrac distance for Principal Coordinate Analysis and Unweighted Pair Group Method with Arithmetic mean.

Exact values of significance are indicated in all figures. All data are presented as means ± *s*.d. in figure legends. Comparisons between two conditions were analysed by unpaired Student’s t-tests. We used GraphPad PRISM version 8.0 to generate graphs and statistics. Differences were considered significant with *p* < 0.05.

## 3 Results

### 3.1 XZP reduced lipid levels in liver and serum of hyperlipidemia hamsters

**FIGURE 1:**
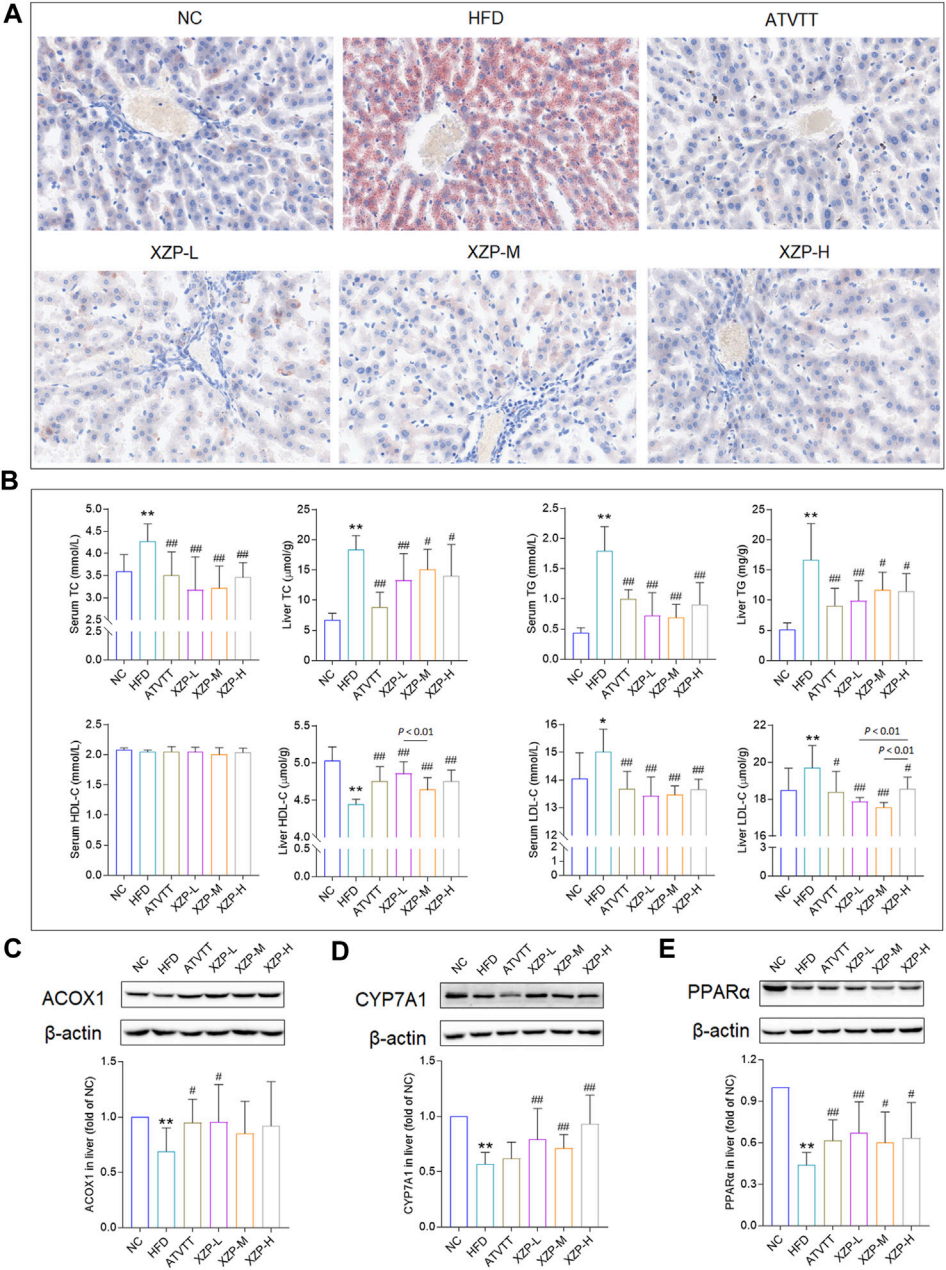
**(A)** Oil red O staining of livers. (Scale bar = 100 μm) **(B)** Lipid index include TC, TG, HDL-C and LDL-C levels in the serum and liver **(C)** Expression of ACOX1 was determined in the liver by WB. **(D)**Expression of CYP7A1 was determined in the liver by WB. **(E)** Expression of PPARα was determined in the liver by WB. *p < 0.05, **p <0.01 *via* HFD group; #*p* < 0.05, ##*p* < 0.01 *via* the NC group.

To determine that hamsters induced by high-fat diet for 3 weeks to hyperlipidemia model, we tested TC and TG of serum from tail tip of hamsters at the end of the induction. TC induced with a high-fat diet was significantly higher than standard diet, and hamsters with lower TC value induced with a high-fat diet were excluded **(** Supplementary Figure S2 **)**. After 4 weeks of XZP and ATVTT intervention, biochemical indexes and liver histopathology were tested. Histochemical examinations using oil red O indicated that XZP and ATVTT could alleviate lipid accumulation in liver **(** Figure 1A **)**. There was no obvious pathological damage was detected in liver tissue of hamsters fed with high-fat diet **(** Supplementary Figure S3 **)**. Both XZP and ATVTT treatment reduced TC, TG and LDL-C in serum and liver of hamsters induced with high-fat diet. Furthermore, XZP and ATVTT treatment could increase HDL-C in liver of hamsters induced with high-fat diet, while not found in serum (Figure 1B; Supplementary Table S1). Acox1 catalyzes the first step in peroxisomal ß-oxidation and enriched in liver. CYP7A1 catalyzes the hydroxylation of cholesterol to 7ahydroxycholesterol. PPARα target genes are involved in fatty acid metabolism in tissues with high oxidative rates such as heart and liver. XZP increased ACOX1, CYP7A1, and PPARα in liver of hamsters induced by high-fat diet **(** Figures 1C–E **)**.

In general, we found that XZP reduced lipid level of blood and liver including TC, TG, LDL-C, and increased the expression of HDL-C in the liver of HFD hamsters. The excessive accumulation of lipid droplets in the liver induced by high fat diet was greatly alleviated by XZP and ATVTT intervention. In addition, XZP It promoted the decomposition and oxidation of fat by increasing the expression of PPARα, ACOX1 and CYP7A1 in liver of hamster induced by high fat diet.

### 3.2 XZP alleviated mild liver injury, oxidative stress and inflammation in hyperlipidemia hamsters

**FIGURE 2:**
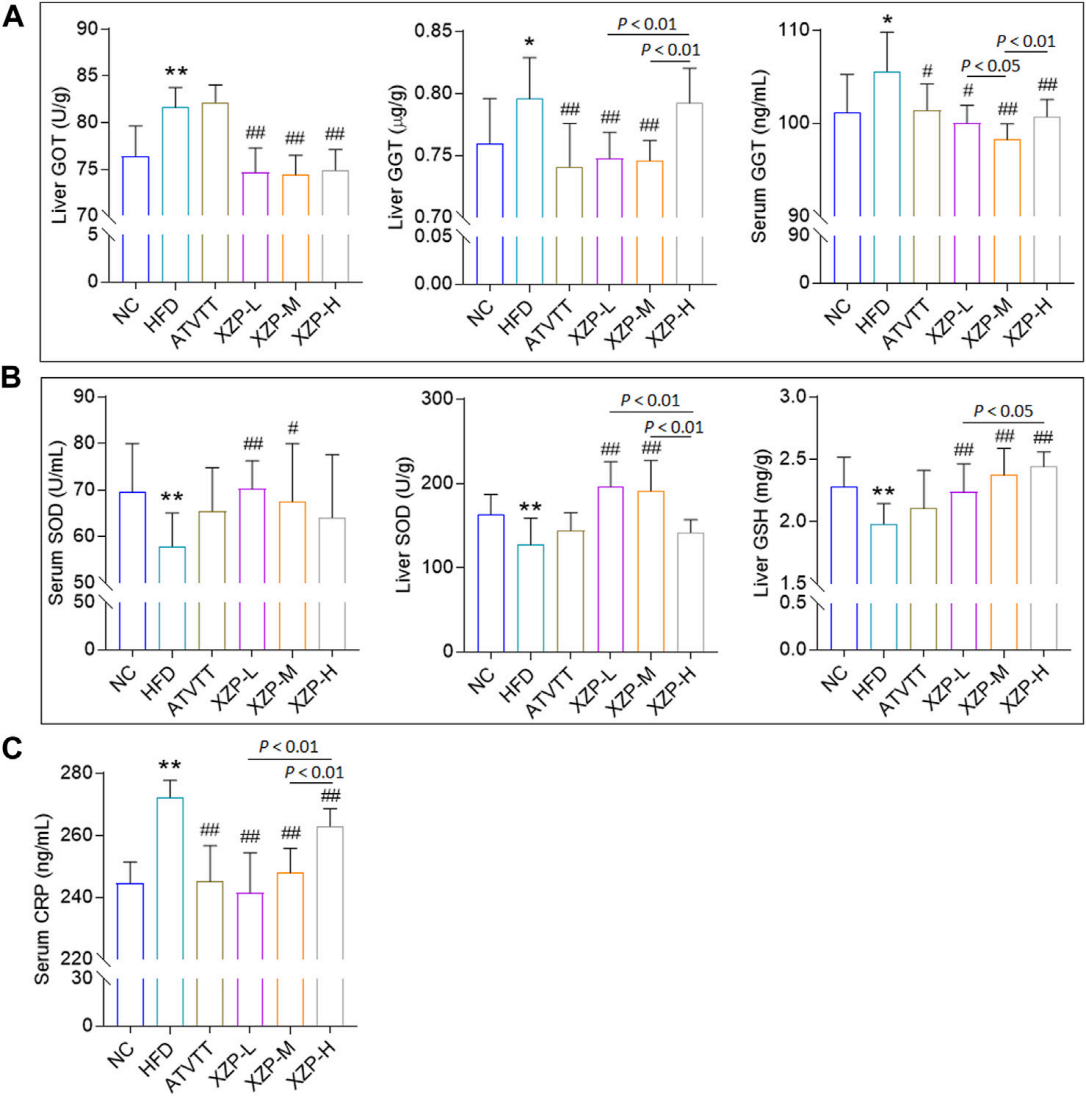
**(A)** Liver function include GOT and GGT **(B)** Antioxidant stress index include SOD and GSH. **(C)** Inflammation index CRP. *p < 0.01, *p < 0.05, **p < 0.01 *via* HFD group; #*p* < 0.05, ##*p* < 0.01 *via* the NC group.

To assess whether XZP and ATVTT can protect against liver damage caused by high fat diet, we examined the liver pathology changes with HE staining and liver function including GOT, GPT, ALP, and GGT in liver and serum **(** Supplementary Table S1 **)**. H&E staining results showed that neither high-fat diet nor drug treatment could cause pathological damage to liver tissue. However, the results showed that XZP and ATVTT reduced GGT in liver and serum. In addition, XZP significantly reduced GOT in liver (Figure 2A). To assess whether XZP and ATVTT can resist oxidative stress, we tested SOD, MDA, and GSH in liver and serum. The results showed that XZP significantly increased SOD in liver and serum, and GSH in liver (Figure 2B). There was no significant difference in the antioxidant stress of ATVTT (Figure 2B). To assess whether XZP and ATVTT can resist inflammation in hamsters induced by high-fat diet, we tested IL-6 and CRP **(** Supplementary Table S1 **)**. The results showed that XZP and ATVTT significantly decreased CRP level in serum (Figure 2C).

The results of this experiment showed that XZP and ATVTT of liver function indicators (GGT and GOT) were remarkably decreased than HFD, which indicated the protective effect on liver function. At the same time, XZP expressed antioxidant stress by increasing SOD and MDA of high fat hamsters.

### 3.3 XZP regulated lipid metabolism in liver, serum and feces

To better understand XZP regulated lipid metabolism of hyperlipidemia hamsters, we measured metabolite profiles of three sample types including serum, liver and feces. The types of lipids mainly include lysophosphatidylcholine (LysoPC), lysophosphatidylserine (LPS), phosphatidylcholine (PC), phosphatidylethanolamine (PE), sphingomyelin (SM), and ceramide (Cer). We evaluated the lipid lowering mechanism of XZP by analyzing the lipid metabolism of serum, liver and feces.

#### 3.3.1 XZP regulated serum lipid metabolism of hyperlipidemia hamsters

**FIGURE 3:**
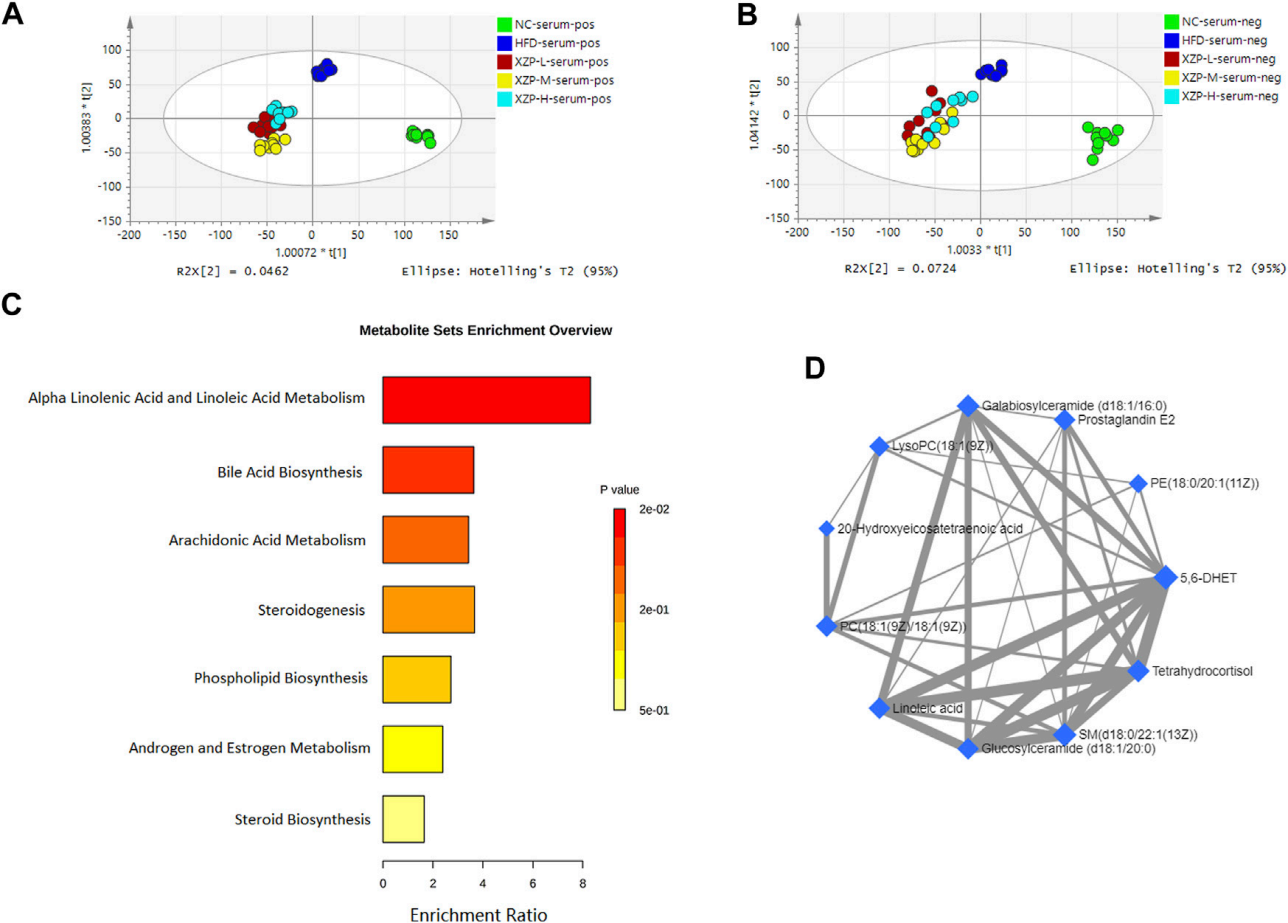
**(A)** OPLS-DA score plots of serum metabolic profiles based molecular features obtained by UPLC-MS/MS [ESI+] **(B)** OPLS-DA score plots of serum metabolic profiles obtained by UPLC-MS/MS [ESI-]. Each dot in the score plots represents an independent sample **(C)** Serum metabolic pathways were enriched by MetaboAnalyst. Enrichment Ratio is computed by Hits/Expected, where hits = observed hits; expected = expected hits. **(D)** Interaction of serum biomarkers were enriched by MetaboAnalyst.

OPLS-DA revealed clear shifts in the serum metabolomic profiles were observed in the HFD compared with NC group, indicated high fat diet changes the serum metabolism of hamsters. Further more, clear shifts in the metabolomic profiles were observed in the XZP-H, XZP-M, and XZP-L group after the intervention compared with HFD group (Figure 3A, B).

We obtained the content information form and identification information form of serum metabolites by standardized processing of Progenesis QI. Then, we selected ions with fragment scores of above 50 and VIP >1 as the identification criteria for compounds with fragment information (Supplementary Table S2). Compared with the NC group, 34 biomarkers in the model group were significantly affected by high fat diet including 9 PC, 4 SM, 2 PE, 4 Cer and 7 LysoPC. Notably, XZP-H, XZP-M, and XZP-L significantly regulated 18, 30 and 33 biomarkers from HFD hamsters respectively (Supplementary Table S3). Thus, XZP-L better regulate the serum lipid metabolism of hamsters induced by high fat diet than other administration groups. On this basis, we enriched serum metabolic pathways and biomarkers by enrichment analysis and network analysis of MetaboAnalyst. We selected the pathway with impact-value above 0.05 for illustration. Linoleic acid and alpha-linolenic acid participated in alpha linolenic acid and linoleic acid metabolism with *p* = 0.0225. Cholic acid participated in bile acid biosynthesis with *p* = 0.0442. Prostaglandin E2 and 5, 6-DHET, 20-hydroxyeicosatetraenoic acid participated in arachidonic acid metabolism with *p* = 0.0414. Tetrahydrocortisol and 11b-hydroxyprogesterone participated in steroidogenesis with *p* = 0.01 (Figure 3C).

The metabolite-metabolite interaction network helps to highlight potential functional relationships between a wide set of annotated metabolites. We found that there was a strong correlation between 5,6-DHET, 20-hydroxyeicosatetraenoic acid, tetrahydrocortisol, linoleic acid, prostaglandin E2, LysoPC(18:1(9Z)), PC(18:1(9Z)/18:1(9Z)), PE(18:0/20:1(11Z)), SM(d18:0/22:1(13Z)), glucosylceramide (d18:1/20:0) and galabiosylceramide (d18:1/16:0) by network analysis of MetaboAnalyst. In addition, we found XZP-H, XZP-M and XZP-L significantly regulated 9, 9 and 10 biomarkers from HFD hamsters respectively (Table 1).

**TABLE 1:** Information on the inter group comparison trend of intensity of enriched serum biomarkers.

NO.	Compound name	HFD*/*NC	XZP-L*/*HFD	XZP-M*/*HFD	XZP-H*/*HFD
1	5,6-DHET	↑ **	↓ #	↓ #	↓ #
2	20-Hydroxyeicosatetraenoic acid	↑ **	↓ #	↓ ##	--
3	Galabiosylceramide (d18:1/16:0)	↑ **	↓ #	↓ #	↓ #
4	Glucosylceramide (d18:1/20:0)	↑ **	↓ #	--	↓ ##
5	Linoleic acid	↑ **	↓ #	↓ #	↓ #
6	LysoPC(18:1(9Z))	↑ **	↓ ##	↓ ##	--
7	PC(18:1(9Z)/18:1(9Z))	↓ **	↑ #	↑ #	↑ #
8	Tetrahydrocortisol	↑ **	—	--	↓ #
9	PE(18:0/20:1(11Z))	↑ **	↓ ##	↓ #	↓ ##
10	Prostaglandin E2	↓ **	↑ ##	↑ ##	↑ ##
11	SM(d18:0/22:1(13Z))	↑ **	↓ #	↓ #	↓ #

* *p* <0.05, **p < 0.01 *via* HFD, group; #*p* < 0.01, ##*p* < 0.001 *via* the NC group.

#### 3.3.2 XZP regulated liver lipid metabolism of hyperlipidemia hamsters

OPLS-DA revealed clear shifts in the liver metabolomic profiles were observed in the HFD compared with NC group, indicated high fat diet changes the liver metabolism of hamsters. Further more, clear shifts in the metabolomic profiles were observed in the XZP-H, XZP-M, and XZP-L group after the intervention compared with HFD group (Figures 3A, B).

**FIGURE 4:**
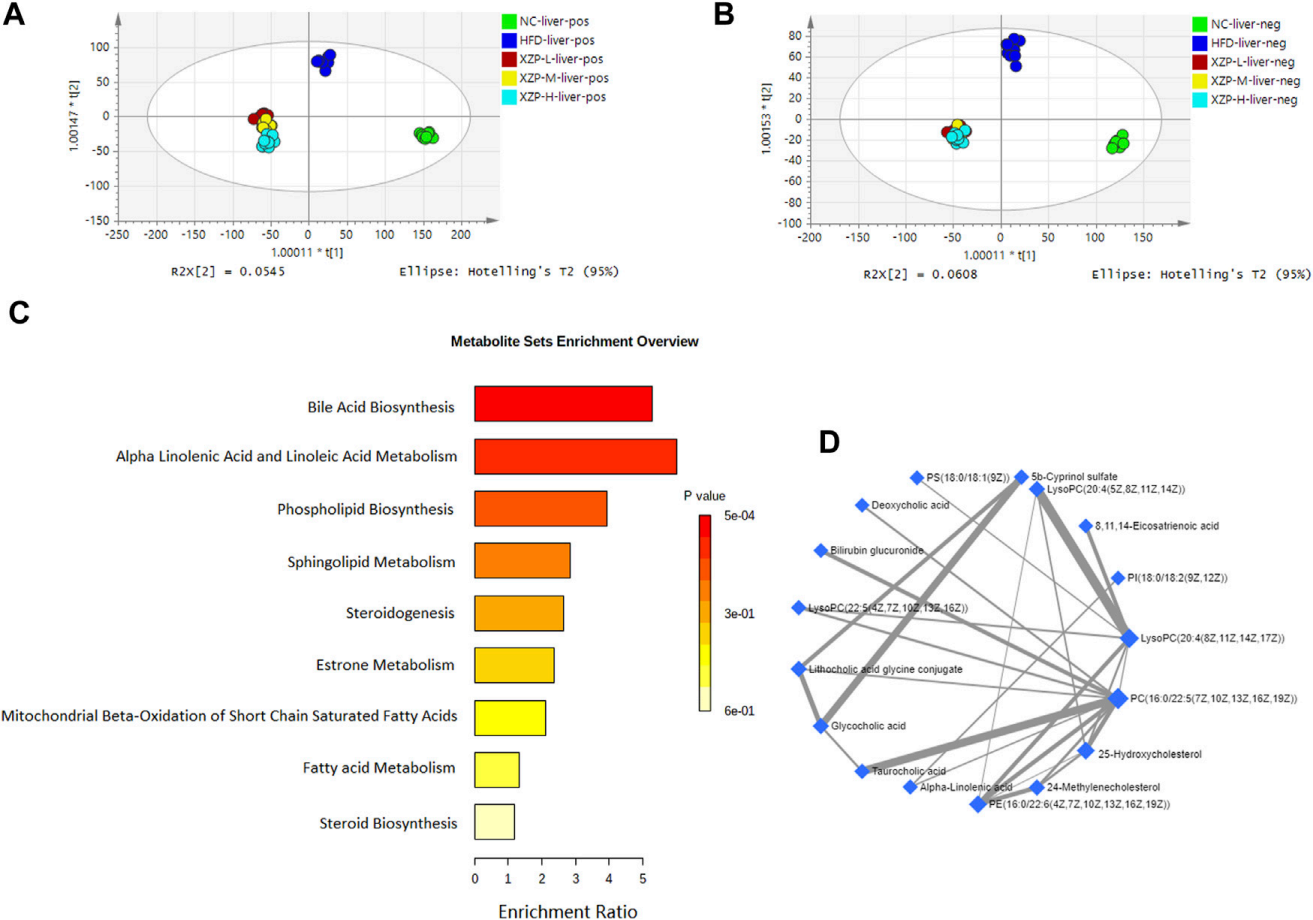
**(A)** OPLS-DA score plots of liver metabolic profiles based molecular features obtained by UPLC-MS/MS [ESI+] **(B)** OPLS-DA score plots of liver metabolic profiles obtained by UPLC-MS/MS [ESI-]. Each dot in the score plots represents an independent sample **(C)** Serum metabolic pathways were enriched by MetaboAnalyst. Enrichment Ratio is computed by Hits/Expected, where hits = observed hits; expected = expected hits. **(D)** Interaction of liver biomarkers were enriched by MetaboAnalyst.

We obtained the content information form and identification information form of liver metabolites by standardized processing of Progenesis QI. Then, we selected ions with fragment scores of above 50 and VIP >1 as the identification criteria for compounds with fragment information (Supplementary Table S4). Compared with the NC group, 52 biomarkers in HFD group were significantly affected by high fat diet including 7 PC, 15 PE, 1 Cer, 12 LysoPC and 2 PI. Notably, XZP-H, XZP-M, and XZP-L significantly regulated 46, 47 and 48 biomarkers from HFD hamsters respectively (Supplementary Table S5). Thus, XZP-L better regulate the liver lipid metabolism of hamsters induced by high fat diet than other administration groups. On this basis, we enriched serum metabolic pathways and biomarkers by enrichment analysis and network analysis of MetaboAnalyst. We selected the pathway with impact-value above 0.05 for illustration. Taurocholic acid, glycocholic acid, deoxycholic acid, lithocholic acid glycine conjugate, and 25-hydroxycholesterol participated in bile acid biosynthesis with *p* = 0.000528. Alpha-linolenic acid, 8,11,14-eicosatrienoic acid participated in alpha linolenic acid and linoleic acid metabolism with *p* = 0.0418. Other liver metabolic pathways include phospholipid biosynthesis, sphingolipid metabolism, steroidogenesis, estrone metabolism, mitochondrial beta-oxidation of short chain saturated fatty acids, fatty acid metabolism and steroid biosynthesis (Figure 4C).

We found that there was a strong correlation between 8,11,14-eicosatrienoic acid, deoxycholic acid, LysoPC(20:4(8Z,11Z,14Z, 17Z)), PI(18:0/18:2(9Z, 12Z)), 5b-cyprinol sulfate, glycocholic acid, PS(18:0/18:1(9Z)), bilirubin glucuronide, taurocholic acid, alpha-inolenic acid, lithocholic acid glycine conjugate, 24-methylenecholesterol, PC(16:0/22:5(7Z,10Z,13Z,16Z, 19Z)), 25-hydroxycholesterol, PE(16:0/22:6(4Z,7Z,10Z,13Z,16Z, 19Z)), LysoPC(22:5(4Z,7Z,10Z,13Z, 16Z)), LysoPC(20:4(5Z,8Z,11Z, 14Z)) by network analysis of MetaboAnalyst. In addition, we found XZP-H, XZP-M and XZP-L significantly regulated 15, 15 and 17 biomarkers from HFD hamsters respectively (Table 2).

**TABLE 2:** Information on the inter group comparison trend of relative abundance of enriched liver biomarkers.

NO.	Compound name	HFD*/*NC	XZP-L*/*HFD	XZP-M*/*HFD	XZP-H*/*HFD
1	5b-Cyprinol sulfate	↓ **	↑ #	↑ ##	↑ #
2	8,11,14-Eicosatrienoic acid	↑ **	↓ #	↓ ##	↓ ##
3	24-Methylenecholesterol	↓ **	↑ #	↑ #	↑ #
4	25-Hydroxycholesterol	↑ **	↑ #	↑ ##	↑ #
5	Alpha-Linolenic acid	↓*	↑ #	--	--
6	Bilirubin glucuronide	↓ **	↑ ##	↑ #	↑ #
7	Deoxycholic acid	↑ **	↓ #	↓ ##	↓ ##
8	Glycocholic acid	↓ **	↑ #	↑ ##	↑ ##
9	Lithocholic acid glycine conjugate	↓ **	↑ ##	↑ #	--
10	LysoPC(20:4(5Z,8Z,11Z,14Z))	↓ **	↑ ##	↑ #	↑ #
11	LysoPC(20:4(8Z,11Z,14Z,17Z))	↑ **	↓ ##	↓ ##	↓ ##
12	LysoPC(22:5(4Z,7Z,10Z,13Z,16Z))	↓ **	↑ ##	↑ #	↑ ##
13	PC(16:0/22:5(7Z,10Z,13Z,16Z,19Z))	↓ **	↑ #	↑ #	↑ ##
14	PE(16:0/22:6(4Z,7Z,10Z,13Z,16Z,19Z))	↓ **	↑ ##	↑ ##	↑ #
15	PS(18:0/18:1(9Z))	↓*	↑ ##	↑ ##	↑ ##
16	PI(18:0/18:2(9Z,12Z))	↓ **	↑ ##	--	↑ #
17	Taurocholic acid	↓ **	↑ ##	↑ ##	↑ #

*p* < 0.05, **p < 0.01 *via* HFD, group; #*p* < 0.01, ##*p* < 0.001 *via* the NC, group.

#### 3.3.3 XZP regulated fecal lipid metabolism of hyperlipidemia hamsters

**FIGURE 5:**
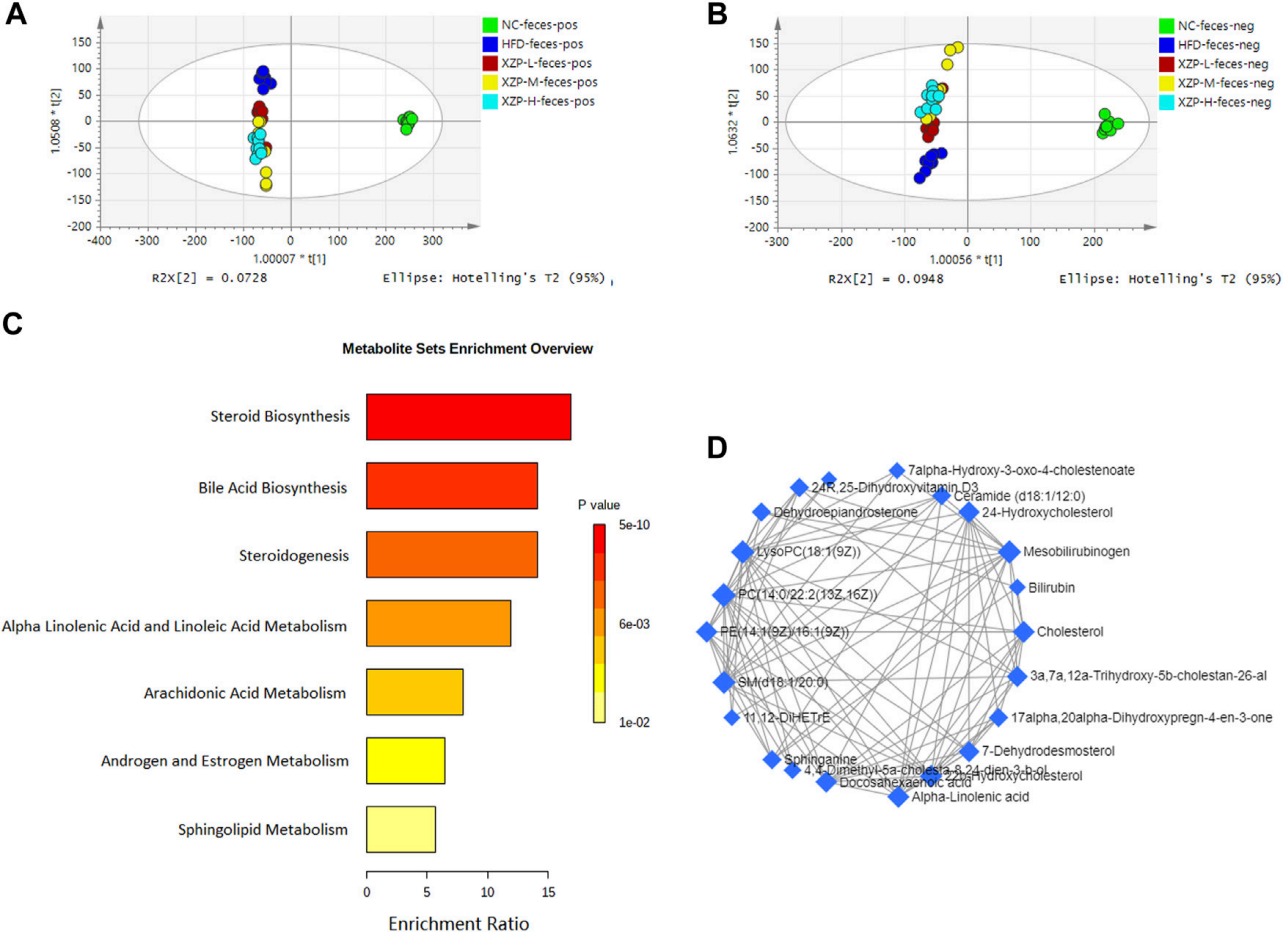
**(A)** OPLS-DA score plots of feces metabolic profiles based molecular features obtained by UPLC-MS/MS [ESI+] **(B)** OPLS-DA score plots of feces metabolic profiles obtained by UPLC-MS/MS [ESI-]. Each dot in the score plots represents an independent sample **(C)** Serum metabolic pathways were enriched by metaboanalyst. Enrichment Ratio is computed by Hits/Expected, where hits = observed hits; expected = expected hits. **(D)** Interaction of feces biomarkers were enriched by MetaboAnalyst.

OPLS-DA revealed clear shifts in the fecal metabolomic profiles were observed in the HFD compared with NC group, indicated high fat diet changes the fecal metabolism of hamsters. Further more, clear shifts in the metabolomic profiles were observed in the XZP-H, XZP-M, and XZP-L group after the intervention compared with HFD group (Figure 5A, B). XZP regulated fecal metabolism of HFD hamsters, but it could not cluster relative to NC.

We obtained the content information form and identification information form of liver metabolites by standardized processing of Progenesis QI. Then, we selected ions with fragment scores of above 50 and VIP >1 as the identification criteria for compounds with fragment information (Supplementary Table S6). Compared with the NC group, 37 biomarkers in the model group were significantly affected by HFD including 7 PC, 3 PE, 4 Cer, 2 SM and 4 LysoPC. Notably, XZP-H, XZP-M and XZP-L significantly regulated 24, 31 and 34 biomarkers from HFD hamsters respectively (Supplementary Table S7). Thus, XZP-L better regulate the liver lipid metabolism of hamsters induced by high-fat diet than other administration groups. On this basis, we enriched fecal metabolic pathways and biomarkers by enrichment analysis and network analysis of MetaboAnalyst. We selected the pathway with impact-value above 0.05 for illustration. Cholesterol, 24-hydroxycholesterol, 3a,7a,12a-trihydroxy-5b-cholestan-26-aL, 3 beta-hydroxy-5-cholestenoate, 7alpha-hydroxy-3-oxo-4-cholestenoate participated in bile acid biosynthesis with *p* < 0.0001. Alpha-linolenic acid and docosahexaenoic acid participated in steroidogenesis (*p* < 0.0001) and alpha linolenic acid and linoleic acid metabolism (*p* < 0.0001). 11,12-DiHETrE participated in arachidonic acid metabolism with *p* = 0.002. Dehydroepiandrosterone participated in androgen and estrogen metabolism with *p* = 0.007. Sphinganine and ceramide (d18:1/18:0) participated in sphingolipid metabolism with *p* = 0.012 (Figure 5C).

We found that there was a strong correlation between cholesterol, 3a,7a,12a-trihydroxy-5b-cholestan-26-aL, 17alpha, 20alpha-dihydroxypregn-4-en-3-one, 7-dehydrodesmosterol, 4,4-dimethyl-5a-cholesta-8,24-dien-3-b-ol, 22b-hydroxycholesterol, docosahexaenoic acid, 24R, 25-dihydroxyvitamin D3, 24-Hydroxycholesterol, 11, 12-DiHETrE, 7alpha-Hydroxy-3-oxo-4-cholestenoate, SM(d18:1/20:0), PE(14:1(9Z)/16:1(9Z)), ceramide (d18:1/12:0), LysoPC(18:1(9Z)), sphinganine, dehydroepiandrosterone, alpha-linolenic acid, 3 beta-hydroxy-5-cholestenoate, PC(14:0/22:2(13Z, 16Z)), mesobilirubinogen and bilirubin by network analysis of MetaboAnalyst. In addition, we found XZP-H, XZP-M and XZP-L significantly regulated 17, 19 and 20 biomarkers from HFD hamsters respectively (Table 3).

**TABLE 3:** Information on the inter group comparison trend of relative abundance of enriched feces biomarkers.

NO.	Compound name	HFD/NC	XZP-L/HFD	XZP-M/HFD	XZP-H/HFD
1	3a,7a,12a-Trihydroxy-5b-cholestan-26-aL	↑ **	--	↓ #	↓ #
2	3 beta-Hydroxy-5-cholestenoate	↓ **	↑ ##	—	↑ #
3	4,4-Dimethyl-5a-cholesta-8,24-dien-3-b-ol	↑ **	↓ ##	↓ ##	↓ #
4	7-Dehydrodesmosterol	↑ **	↓ #	↓ ##	↓ ##
5	7alpha-Hydroxy-3-oxo-4-cholestenoate	↑ **	↓ ##	↓ ##	↓ #
6	11,12-DiHETrE	↑ **	↓ #	↓ ##	↓ ##
7	17alpha,20alpha-Dihydroxypregn-4-en-3-one	↑ **	↓ #	↓ #	-
8	22b-Hydroxycholesterol	↑ **	↓ #	↓ #	↓ ##
9	24-Hydroxycholesterol	↑ **	↓ ##	↓ ##	↓ ##
10	24R,25-Dihydroxyvitamin D3	↑ **	↓ ##	↓ ##	↓ ##
11	Alpha-Linolenic acid	↓ **	↑ #	↑ ##	--
12	Bilirubin	↓ **	↑ #	↑ #	↑ #
13	Ceramide (d18:1/12:0)	↑ **	↓ ##	↓ #	↓ #
14	Cholesterol	↑ **	↓ #	--	--
15	Dehydroepiandrosterone	↓ **	↑ ##	↑ ##	↑ ##
16	Docosahexaenoic acid	↑ **	--	↓ ##	↓ #
17	LysoPC(18:1(9Z))	↑ **	↓ #	↓ #	--
18	Mesobilirubinogen	↑ **	↑ #	↑ #	↑ ##
19	PC(14:0/22:2(13Z,16Z))	↓ **	↑ ##	↑##	↑ ##
20	PE(14:1(9Z)/16:1(9Z))	↑ **	↓ #	↓ #	↓ #
21	SM(d18:1/20:0)	↑ **	↓ ##	↓ ##	↓ ##
22	Sphinganine	↓ **	↑ ##	—	—

*p* < 0.05, **p < 0.01 *via* HFD, group; #*p* < 0.05, ##*p* < 0.01 *via* the NC, group.

**FIGURE 6:**
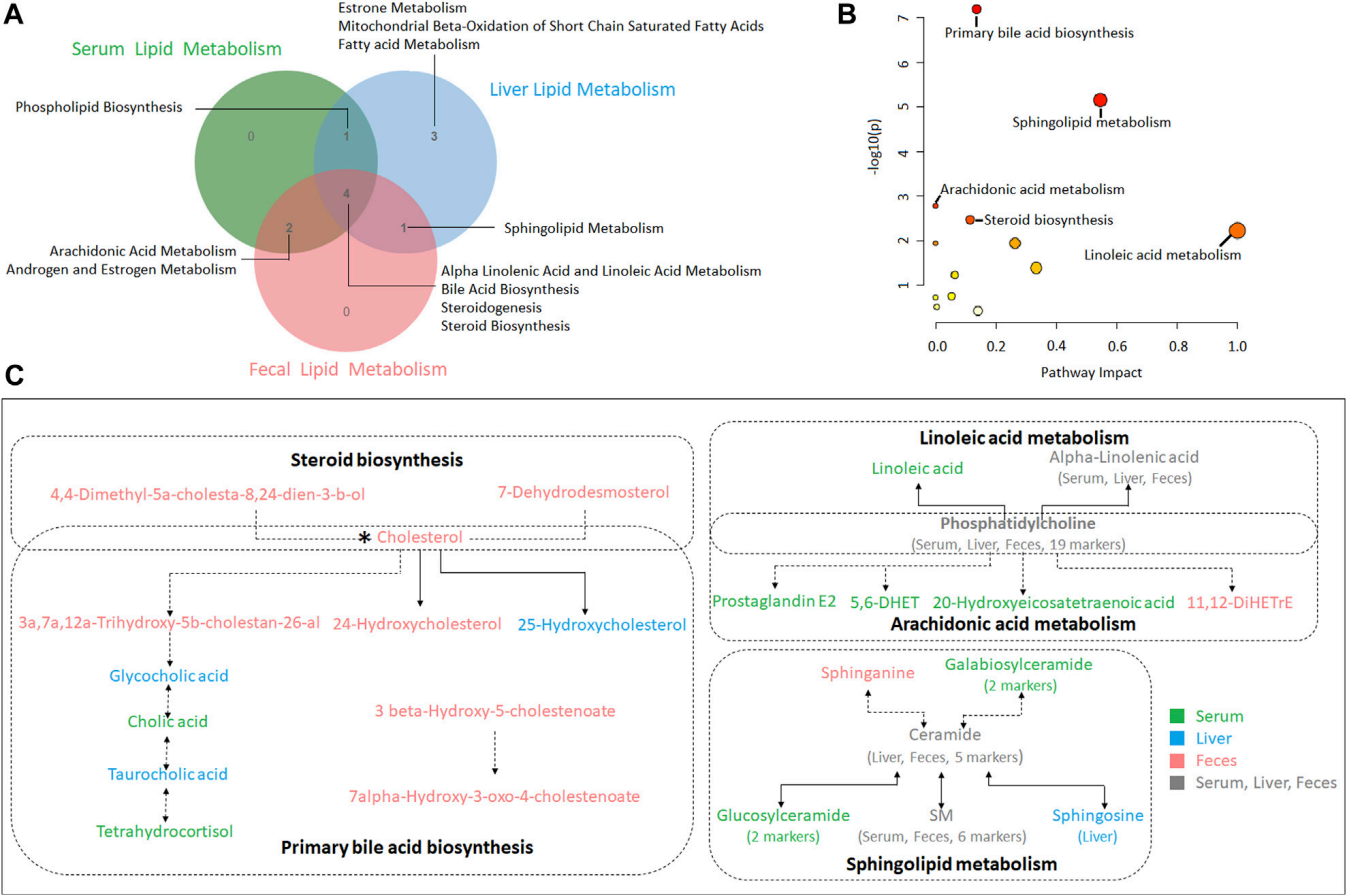
**(A)** A Venn diagram showing shared or unique lipid metabolism pathway among serum, liver and feces samples **(B)** Pathway analysis of serum, liver and feces biomarkers with MetPA. **(C)** The correlation among the biomarkers participating in metabolic pathways of hyperlipidemia regulated by XZP.

In the present study, serum, liver and feces metabolism shared four pathways including alpha linoleic acid and linoleic acid metabolism, bile acid biosynthesis, steroidogenesis and steroid biosynthesis **(** Figure 6A). MetPA analysis of all biomarkers in serum, liver and feces showed that the five metabolic pathways with significance from high to low were primary bile acid biosynthesis, sphingolipid metabolism, arachidonic acid metabolism, steroid biosynthesis and linoleic acid metabolism **(** Figure 6B; Supplementary Table S8 **).** The correlation among the biomarkers showed that cholesterol was the key marker between primary bile acid biosynthesis and steroid biosynthesis, and phosphatidylcholine identified in serum, liver and feces were the key marker between arachidonic acid metabolism and linoleic acid metabolism. In addition, ceramide identified in liver and feces the key marker of sphingolipid metabolism **(** Figure 6C **)**.

### 3.4 XZP regulated gut microbiota composition of hyperlipidemia hamsters

**FIGURE 7:**
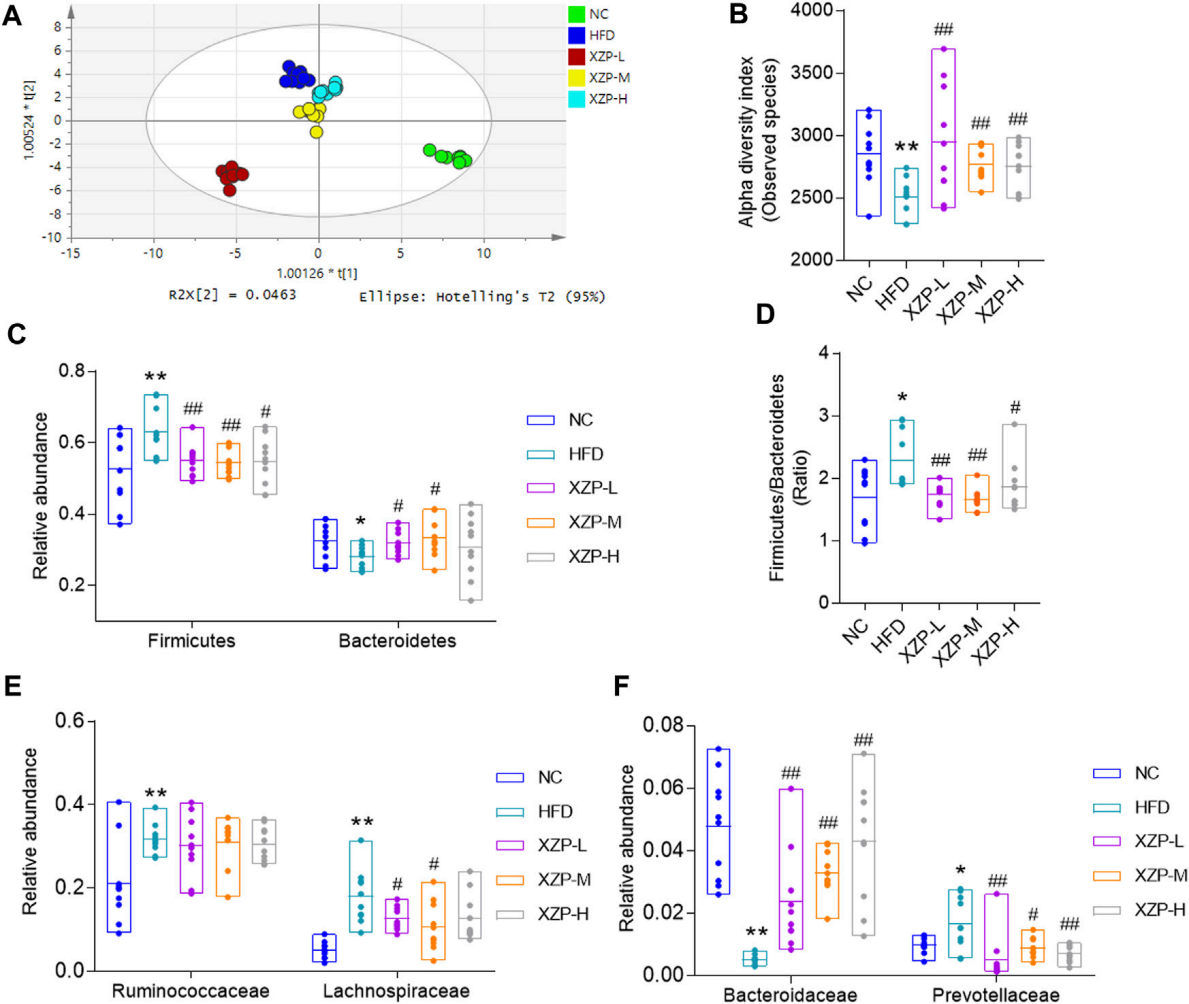
**(A)** OPLS-DA score plots based on genus profiles obtained by 16S rRNA sequencing **(B)** Alpha -diversity of gut microbiota calculated by observed species. **(C)** Relative abundance of Firmicutes and Bacteroidetes **(D)** Ratio of relative abundance between Firmicutes and Bacteroidetes. **(E)** Relative abundance of Ruminococcaceae and Lachnospiraceae genes in Firmicutes **(F)** Relative abundance of Bacteroidaceae and Prevotellaceae in Bacteroidetes. *p < 0.05, **p < 0.01 *via* HFD group; #*p* < 0.05, ##*p* < 0.01 *via* the NC group.

The overall structural changes of gut microbiota in response to XZP were determined by analysis of the 16S rRNA gene sequences of microbial samples isolated from the feces of all groups. OPLS-DA revealed distinct clustering of intestinal microbe genus for each experimental group. The microbes in XZP-L, XZP-M, and XZP-H were significantly away from HFD group, which is an indication that XZP regulated gut microbiota of hamsters induced by high fat diet (Figure 7A). Alpha-diversity of gut microbiota calculated by observed species. We found that high fat diet significantly reduced alpha-diversity of gut microbiota of hamsters. XZP significantly increased the alpha-diversity of gut microbiota induced by high fat diet (Figure 7B). Firmicutes and Bacteroidetes are the main composition of hamsters. The results showed that high fat diet significantly increased the relative abundance of Firmicutes and decreased the relative abundance of *Bacteroides*. Each group of XZP significantly decreased the relative abundance of Firmicutes in hamsters induced by high fat diet. However, XZP-M and XZP-L significantly the relative abundance of *Bacteroides* in hamsters induced by high fat diet (Figure 7C). High fat diet significantly increased the ratio of Firmicutes and Bacteroidetes (F/B) of hamsters, which was significantly decreased by each group of XZP (Figure 7D). The relative abundance of Ruminococcaceae and Lachnospiraceae genes in Firmicutes both significantly increased in faces of HFD. XZP-M, XZP-L significantly decreased the relative abundance of Lachnospiraceae (Figure 7E). The relative abundance of Bacteroidaceae family in Bacteroidetes significantly decreased, while Prevotellaceae significantly increased in HFD. Each group of XZP significantly regulated Bacteroidaceae and Prevotellaceae (Figure 7F).

**FIGURE 8:**
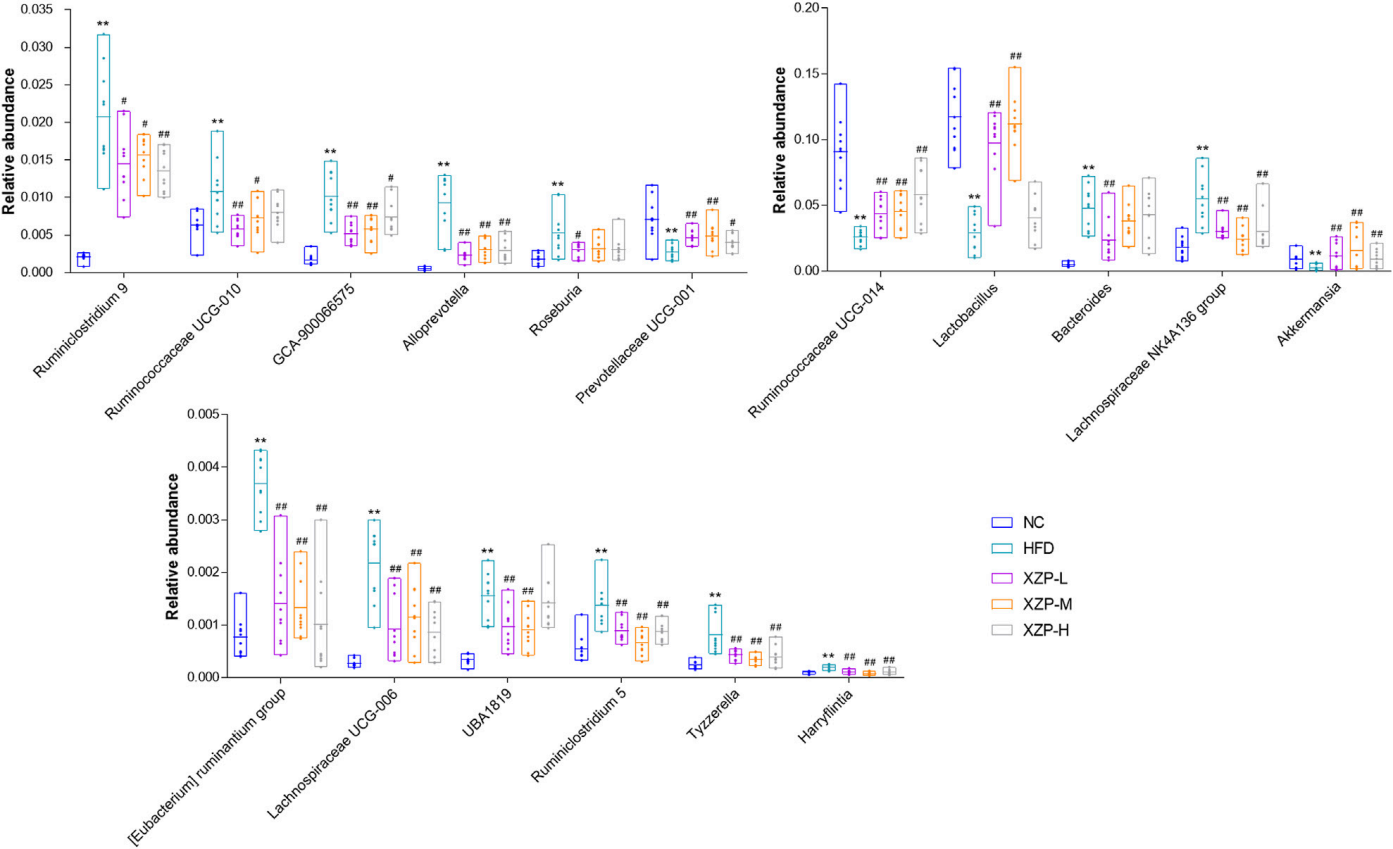
Gut microbiota was determined by 16s rRNA sequencing. Bar charts of fecal microbiota genus composition. *p < 0.05, **p < 0.01 *via* HFD group; #*p* < 0.05, ##*p* < 0.01 *via* the NC group.

The results show that Firmicutes and Bacteroidetes are the main composition of each group. The ratio of Firmicutes and Bacteroidetes was closely related to hyperlipidemia. Therefore, we focused on the analysis of the genus of Firmicutes and Bacteroidetes. We found that Ruminococcaceae UCG-014, Ruminiclostridium 9 and *Lactobacillus* genus of Firmicutes significantly decreased in HFD than NC, while Ruminococcaceae UCG-010, Ruminiclostridium 5, UBA 1819, Harryflintia, Lachnospiraceae NK4A136 group, Roseburia, GCA-900066575 [Eubacterium] ruminantium group, Lachnospiraceae UCG-006 and Tyzzerella genus of Firmicutes significantly increased in HFD than NC. *Bacteroides* and Prevotellaceae UCG-001 genus of Bacteroidetes significantly decreased in HFD than NC, while Alloprevotella genus significantly increased in HFD than NC. Accumulating evidence points to Akkermansia muciniphila as a novel candidate to prevent or treat obesity-related metabolic disorders. We observed hat high fat diet significantly reduced Akkermansia, which could be relieved after oral administration. Finally, we found that XZP-H, XZP-M and XZP-L significantly regulated 12, 15 and 17 the genus of gut microbiota of hamsters induced by high fat diet respectively (Figure 8).

**FIGURE 9:**
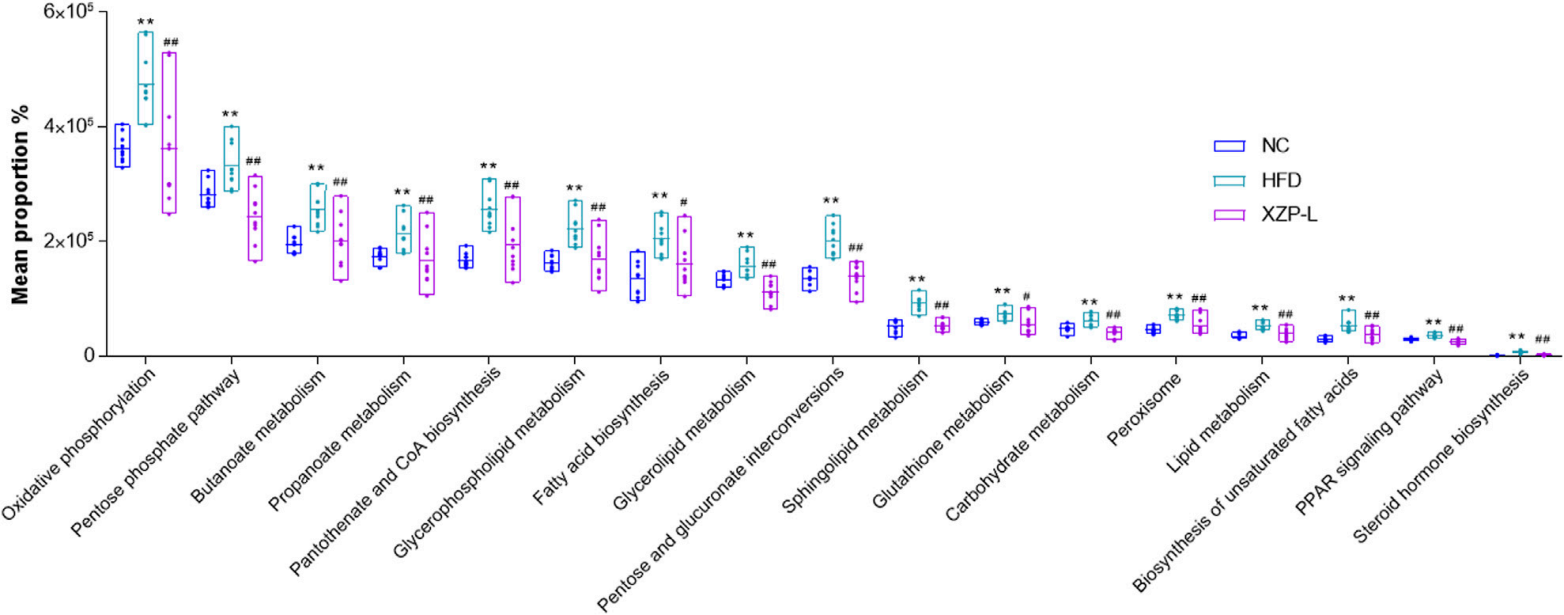
Bioinformatics pathways of fecal microbiota in NC, HFD and XZP-L groups were analyzed. **p < 0.01 *via* HFD group; #*p* < 0.05, ##*p* < 0.01 *via* the NC group.

**FIGURE 10:**
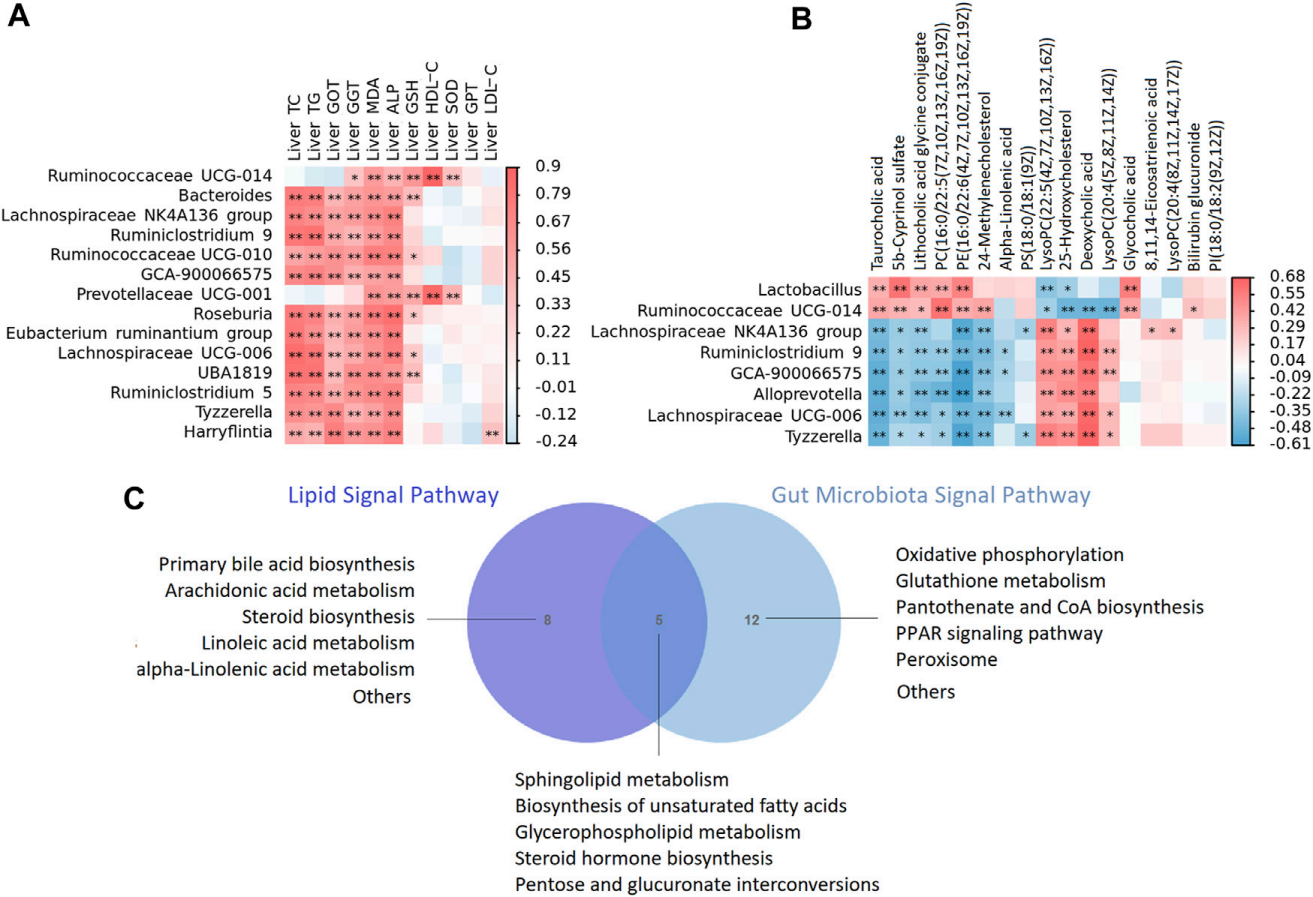
**(A)** The heat map depicts relationships between the genus of microbiota and enriched metabolites of liver **(B)** The heat map depicts relationships between the genus of microbiota and phenotypes of liver including liver lipid, liver function, anti oxidative stress and anti inflammatory indicators. The correlations are determined by Spearman correlation test subject. The legend shows the value of correlation coefficient, red represents positive correlation and blue represents negative correlation. **p < 0.01, *p < 0.05. **(C)** A Venn diagram showing shared or unique lipid signal pathway among lipid and gut microbiota.

We used PICRUSt software to infer the functional gene composition of samples by comparing the species composition information obtained from 16S sequencing data, so as to analyze the functional differences between different groups. XZP-L was better distinguish from HFD than XZP-H and XZP-M, therefore, we take XZP-L as an example to enrich gut microbiota signal pathway. KEGG function prediction showed that high fat diet significantly increased the lipid metabolism related pathways involved by intestinal bacteria including glycerophospholipid metabolism, fatty acid biosynthesis, biosynthesis of unsaturated fatty acids, sphingolipid metabolism, steroid hormone biosynthesis and glycerolipid metabolism, and carbohydrate metabolism including oxidative phosphorylation, pentose and glucuronate interconversions and pentose phosphate pathway (Figure 9). Both lipid metabolism and carbohydrate metabolism are involved in energy metabolism of hamsters, which indicated XZP increase energy consumption by regulating microbiota of hamsters induced by high fat diet. XZP regulated gut microbiota to participate in peroxisome, PPAR signaling pathway and pantothenate and CoA biosynthesis, which related to the synthesis and degradation of fatty acids. In addition, we found that XZP regulates intestinal flora to participate in short chain fatty acid metabolism including butanoate metabolism and propanoate metabolism. Short chain fatty acid metabolism plays an important role in maintaining the normal function of the large intestine and the morphology and function of colon epithelial cells. Notably, XZP regulates gut microbiota to participate in glutathione metabolism, which indicated that XZP enhanced antioxidant stress in high fat diet hamsters by regulating intestinal flora (Figure 10). In conclusion, the gut microbiota analysis showed that XZP increased diversity index and the ratio of the phyla Firmicutes and Bacteroidetes in high fat diet hamsters, which promoted decomposition and oxidation of fatty acids for decreasing the accumulation of lipid.

### 3.5 XZP regulated gut-liver axis of hyperlipidemia hamsters

To confirm the strong link between fecal microbes and fecal metabolites, we performed correlation analysis to examine the associations between the differentially abundant genera and metabolites. In general, we observed strong negative associations between Lachnospiraceae NK4A136 group, Ruminiclostridium 9, GCA-900066575, Alloprevotella, Lachnospiraceae UCG-006, Tyzzerella and taurocholic acid, 5b-cyprinol sulfate, lithocholic acid glycine conjugate, PC(16:0/22:5(7Z,10Z,13Z,16Z, 19Z)), PE(16:0/22:6(4Z,7Z,10Z,13Z,16Z, 19Z)); while strong positive associations between the above microbiota and LysoPC(22:5(4Z,7Z,10Z,13Z, 16Z)), 25-hydroxycholesterol, deoxycholic acid, LysoPC(20:4(5Z,8Z,11Z, 14Z)). In addition, *Lactobacillus*, we observed strong negative associations between Ruminococcaceae UCG-014 and 25-hydroxycholesterol, deoxycholic acid, LysoPC(20:4(5Z,8Z,11Z, 14Z)), while strong positive associations between the above microbiota and taurocholic acid, 5b-cyprinol sulfate, lithocholic acid glycine conjugate, PC(16:0/22:5(7Z,10Z,13Z,16Z, 19Z)), PE(16:0/22:6(4Z,7Z,10Z,13Z,16Z, 19Z)) (Figure 10A).

We then tested the relationship between the genus of microbiota and phenotypes indicators of liver. In general, we observed strong positive associations between *Bacteroides*, Lachnospiraceae NK4A136 group, Ruminiclostridium 9, GCA-900066575, Prevotellaceae UCG-001, Roseburia [Eubacterium] ruminantium group, Lachnospiraceae UCG-006, UBA 1819, Tyzzerella, Harryflintia and phenotypes of liver including liver TC, TG, GOT, GGT, ALP, MDA and GSH (Figure 10B).

In the present study, lipid metabolism and gut microbiota shared five signal pathways including sphingolipid metabolism, biosynthesis of unsaturated fatty acids, glycerophospholipid metabolism, steroid hormone biosynthesis, pentose and glucuronate interconversions **(** Figure 10C **)**. Gut microbiota participated in oxidative phosphorylation, glutathione metabolism, pantothenate and CoA biosynthesis, PPAR signaling pathway and peroxisome, which is highly related to lipid metabolism. The results of correlation analysis showed that XZP regulated expressions of lipid and the antioxidant stress substances in the liver by affecting gut microbiota, although their relationship is hard to explain.

## 4 Discussion

In this study, we established a hyperlipidemia model by feeding with high fat diet for 3 weeks. The results revealed that XZP treatment could reduce TC, TG and LDL-C in serum and liver, while increased HDL-C in liver of hamsters. XZP alleviated lipid accumulation in liver. Although we found that XZP reduced GGT and GOT in serum and liver, no obvious pathological damage was detected in liver tissue of hamsters fed with high-fat diet. This result may be due to the short duration of induction with high fat diet for 7 weeks, and only the accumulation of lipid in liver without hepatocyte damage. In particular, XZP increased SOD in serum and liver, and GSH in serum of hamsters induced by high fat diet. XZP increased the expression of CYP7A1 in liver, which promoted the rate of bile acid biosynthesis (Zhang et al., 2022). Therefore, we speculate that the lipid-lowering effect of XZP may be closely related to its antioxidant stress, and try to find the relationship from lipid metabolism and gut microbiota.

We found XZP regulated alpha linolenic acid and linoleic acid metabolism in serum, liver and feces, alpha-linolenic and linoleic acid in serum, alpha-linolenic acid and 8,11,14-eicosatrienoic acid in liver, alpha-linolenic acid and docosahexaenoic in feces all participate in alpha linolenic acid and linoleic acid metabolism. Therefore, we found that alpha-linolenic was the focus of alpha linolenic acid and linoleic acid metabolism. Our experimental results show that XZP-L significantly increased the level of alpha linolenic acid in serum, liver and feces of hamster fed with high fat diet. The researchers systematically studied investigated the effects of alpha-linolenic acid on the body composition, liver weight, glucose homeostasis, hepatic cholesterol levels, metabolic endotoxemia and systemic inflammation, white adipose tissue homeostasis, liver homeostasis, intestinal homeostasis, and gut microbiota of HFD animals, and found that *a*-linolenic acid administration significantly improved the host metabolic phenotype and gut microbiota of mice fed a high-fat diet, and there was a correlation between the improved gut microbiota and metabolic phenotype (Goncalves et al., 2018; Gao et al., 2020). Alpha-linolenic acid significantly promoted mitochondrial biogenesis, enhanced mitochondrial fatty acid oxidation capacity, improved mitochondrial dynamics, and restored mitochondrial membrane potential, reduced ROS production in the liver tissue of HFD mice (Han et al., 2021). Alpha-linolenic acid further increased the expression of ACOX1-associated proteins and suppressed PPARα-induced proteins relative to HFD (Liput et al., 2021; O'Reilly et al., 2020).

Bile acid metabolism is a lipid metabolism pathway enriched in liver, serum and fecal metabolism. High-fat diet caused hyperlipidemia, which worsened disturbances in bile acid metabolism and gut microbiota (Duan et al., 2021). In serum metabolism, cholic acid participated in regulating bile acid metabolism. In liver metabolism, six biomarkers taurocholic acid, glycocholic acid, deoxycholic acid, lithocholic acid glycine conjugate, 3a, 7a-dihydroxy-5b-cholestan-26-aL and 7 alpha, 24-dihydroxy-4-cholesten-3-one in regulating bile acid metabolism. In fecal metabolism, five biomarkers including cholesterol, 24-hydroxycholesterol, 3a,7a, 12a-trihydroxy-5b-cholestan-26-aL, 3 beta-hydroxy-5-cholestenoate and 7alpha-hydroxy-3-oxo-4-cholestenoate participated in regulating bile acid metabolism. Therefore, we found that XZP regulates the feces and liver bile acid metabolism of high-fat diet hamsters, which was not obvious in serum. High fat diet had a much greater impact on gut microbiota composition including Parabacteroides, *Bacteroides* and *Flavobacterium* genera, increased intestinal permeability and destroyed bile acid homeostasis (Carbajo-Pescador et al., 2019; Rohr et al., 2020).

Arachidonic acid metabolism was enriched in serum and feces metabolism, which was not found in liver metabolism. 11,12-DiHETrE in feces, Prostaglandin E2, 5,6-DHET and 20-hydroxyeicosatetraenoic acid in serum participated in regulating arachidonic acid metabolism. Therefore, we found that arachidonic acid metabolic pathway was the most significant in fecal metabolism. Arachidonic acid, a kind of fatty acid belongs to inflammation biomarker related to deposition of excess fat. Research shows that HFD increased inflammatory enzyme expression, elevated lipid peroxidation product content and oxidative system impairment (Sztolsztener et al., 2020). The study found that intraperitoneal glucose injection induces changes in hypothalamic distribution and amounts of phospholipids, especially arachidonic-acid-containing phospholipids, then metabolized to produce prostaglandins, while continuous activation of the same pathway to produce prostaglandins during HFD deteriorates glucose metabolism (Zhuang et al., 2017; Lee et al., 2021). Arachidonic acid exacerbated NAFLD along with amplified inflammation through TLR4-NF-κB pathway, while alleviated obesity-related disorders *via* rescuing anti-inflammatory and butyrate-producing microbiota, up-regulating GPR41 and GPR109A and controlling hypothalamic inflammation in female (Zhuang et al., 2017). Farnesoid X Receptor (FXR) activated arachidonic acid metabolism in the liver of mice induced by high-fat diet and NF-kB signaling (Gai et al., 2018).

Studies have shown that long-term fed with high fat diet may alter gut microbiota, induce intestinal barrier dysfunction, and hence promote chronic inflammation that contributes to disrupted glycemic homeostasis (He et al., 2020; Ahrens et al., 2021; Cheng et al., 2021). GP as one of the constituent drugs of XZP, reduced the Firmicutes/Bacteroidetes ratio (Li S. et al., 2022), enriched the abundance of Lactococcus spp. and inhibiting the abundance of Ruminococcus spp. in the gut (Shen et al., 2020). Our research showed that, XZP-L effectively regulated the gut microbiota induced by high fat diet and distinguished from HFD group. Akkermansia muciniphila reduced non-esterified fatty acids and energy metabolism (Anhê et al., 2019; Liao et al., 2022), improved glucose homeostasis (Yoon et al., 2021), decreased serum TG and maintains gut homeostasis in HFD-induced animals (Kim et al., 2020). Besides, Akkermansia muciniphila and its derivates had anti-inflammatory properties in liver injury of HFD/CCL4-induced murine model (Raftar et al., 2022). Our research show that XZP-L increased the relative abundance of Akkermansia in hamster feces induced by high-fat diet. Prevotella was more abundant in subjects with a high inflammatory index (Aranaz et al., 2021). Prevotellaceae UCG-004 increased production of the butyric acid significantly upregulated the metabolism of ascorbate and aldarate metabolism, thereby improving the antioxidant properties of Hu sheep (Li C. et al., 2022). In our study, XZP increased the relative abundance of Prevotellaceae UCG-001 genus in hamster feces induced by high fat diet.

Clinical research indicated that relative abundance of Ruminococcaceae and diversification richness highly correlated with TC and TG (Weaver et al., 2018). We found eight genera illustrating strong correlations with liver lipid metabolism. Ruminococcaceae in most inflammatory bowel diseasepatients is abundant (Wang et al., 2021). In the present study, Ruminiclostridium 5, Ruminiclostridium 9, and Ruminococcaceae UCG-010 significantly increased in HFD than NC, while Ruminococcaceae UCG-014 significantly decreased, and XZP regulated the gut microbiota composition of Ruminococcaceae family. We found fourteen genera illustrating strong correlations with phenotypic indicators in the liver. Gut microbiota confers host resistance to obesity by metabolizing dietary polyunsaturated fatty acids (Yu et al., 2019b; Miyamoto et al., 2019). However, we cannot determine the causal relationship between bacterial flora and lipid metabolism.

**FIGURE 11:**
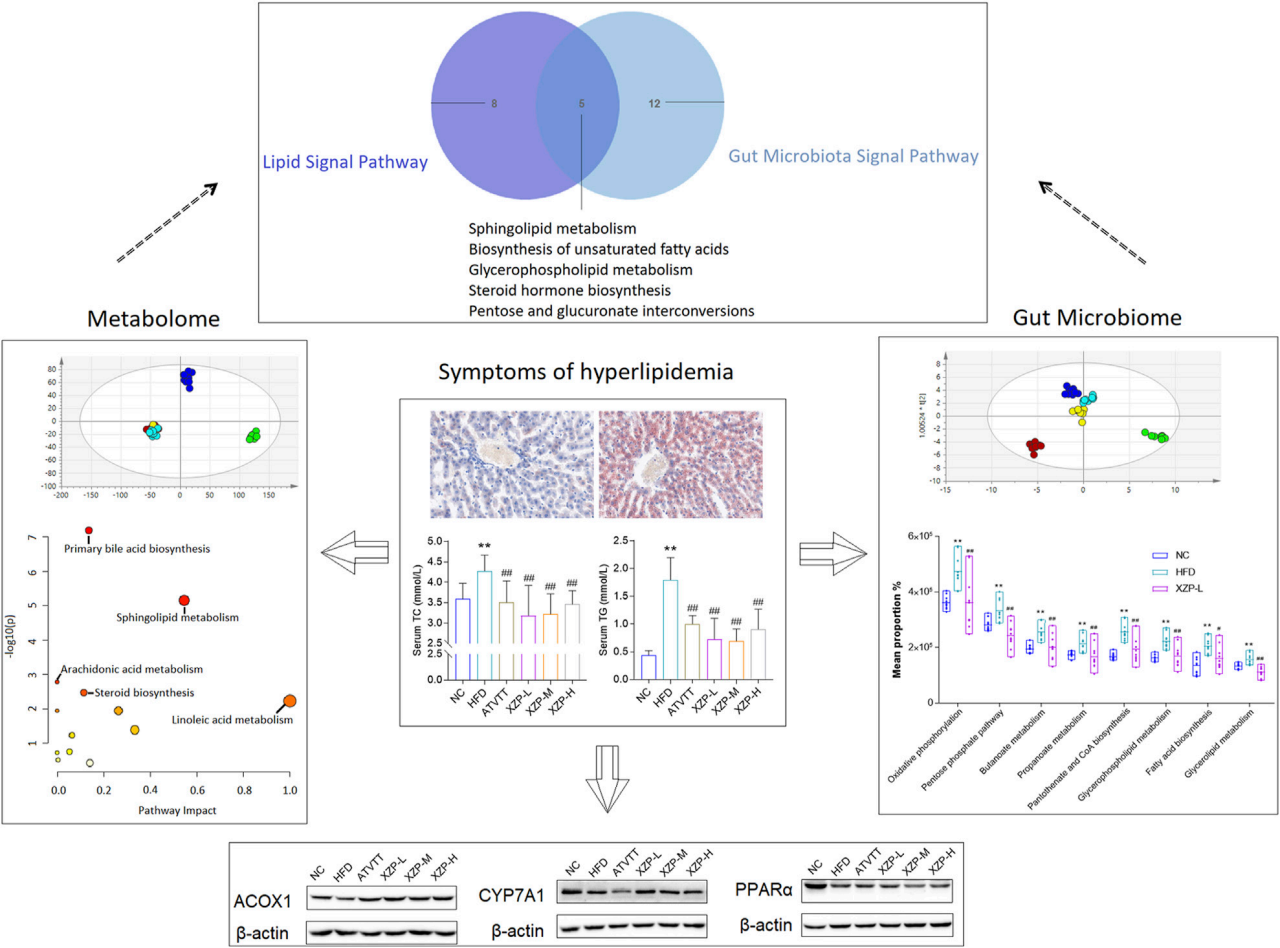
The highlights of the study are illustrated in graphical abstract: XZP reduced lipid levels in liver and serum of hyperlipidemia hamsters; XZP alleviated mild liver injury, oxidative stress and inflammation in hyperlipidemia hamsters; XZP regulated lipid metabolism liver, serum and feces may be related to gut microbiota.

In conclusion, our study confirmed that XZP reduced blood lipid and liver lipid, protected liver function, anti inflammation and anti-oxidation in high-fat diet hamsters. XZP significantly regulated the lipid metabolism in serum and liver of HFD hamsters, including alpha linolenic acid and linoleic acid metabolism, bile acid biosynthesis and arachidonic acid metabolism. XZP reduced the ratio of Firmicutes to *Bacteroides* in high-fat induced hamster faces and reconstructed gut microbiota. There were eight genera illustrating strong correlations with liver lipid metabolism, and fourteen genera illustrating strong correlations with phenotypic indicators in the liver. Gut microbiota regulated by XZP participated in lipid metabolism and oxidative stress signal pathways including glutamate metabolism, peroxisome, PPAR signaling pathway and pantothenate and CoA biosynthesis (Figure 11).

## Data Availability

The datasets presented in this study can be found in online repositories. The names of the repository/repositories and accession number(s) can be found in the article/Supplementary Material.

## Abbreviations

ACOX1, acetyl CoA carboxylase 1; AKP, alkaline phosphatase; Cer, ceramide; CRP, C-reactive protein; CYP7A1, cholesterol 7-alpha hydroxylase; FRR, Fructus Rosa Roxburghii; GGT, gamma glutamyl transferase; GOT, glutamic oxaloacetic transaminase; GP, Gynostemma pentaphyllum; GPT, glutamic pyruvic transaminase; GSH, glutathione; Hawthorn, Crataegus pinnatifida Bge; HDL-C, high density liptein cholesterol; HFD, high-fat diet; LDL-C, low density Lipoprotein; LPS, lysophosphatidylserine; LysoPC, lysophosphatidylcholine; MDA, malondialdehyde; MetPA, metabolomic pathway analysis; OPLS-DA, orthogonal partial least squares discriminant analysis; PC, phosphatidylcholine; PE, phosphatidylethanolamine; PPARα, peroxisome proliferators-activated receptors *a*; SM, sphingomyelin; SOD, superoxide dismutase; TC, total cholesterol; TG, triglyceride; VIP, variable importance; XZP, Xuezhiping capsule.
