# Supplementary material for: Metabolome combined with gut microbiome revealed the lipid-lowering mechanism of Xuezhiping capsule on hyperlipidemic hamster induced by high fat diet
Source: Front Mol Biosci. 2023 Feb 20;10:1147910. doi: 10.3389/fmolb.2023.1147910 (PMC9986548; doi:10.3389/fmolb.2023.1147910)
Supplement: Supplementary file 1 [file Table1.docx]

Supplementary Material

Metabolome Combined with Gut Microbiome Revealed the Lipid-lowering Mechanism of Xuezhiping Capsule on Hyperlipidemic Hamster Induced by High Fat Diet

**Li Wang, Zhixin Zhang, Gan Luo, Ying Wang, Ke Du, Xiaoyan Gao*.**

**Correspondence:** Xiaoyan Gao, [gaoxiaoyan@bucm.edu.cn](mailto:gaoxiaoyan@bucm.edu.cn)

# Supplementary Figures


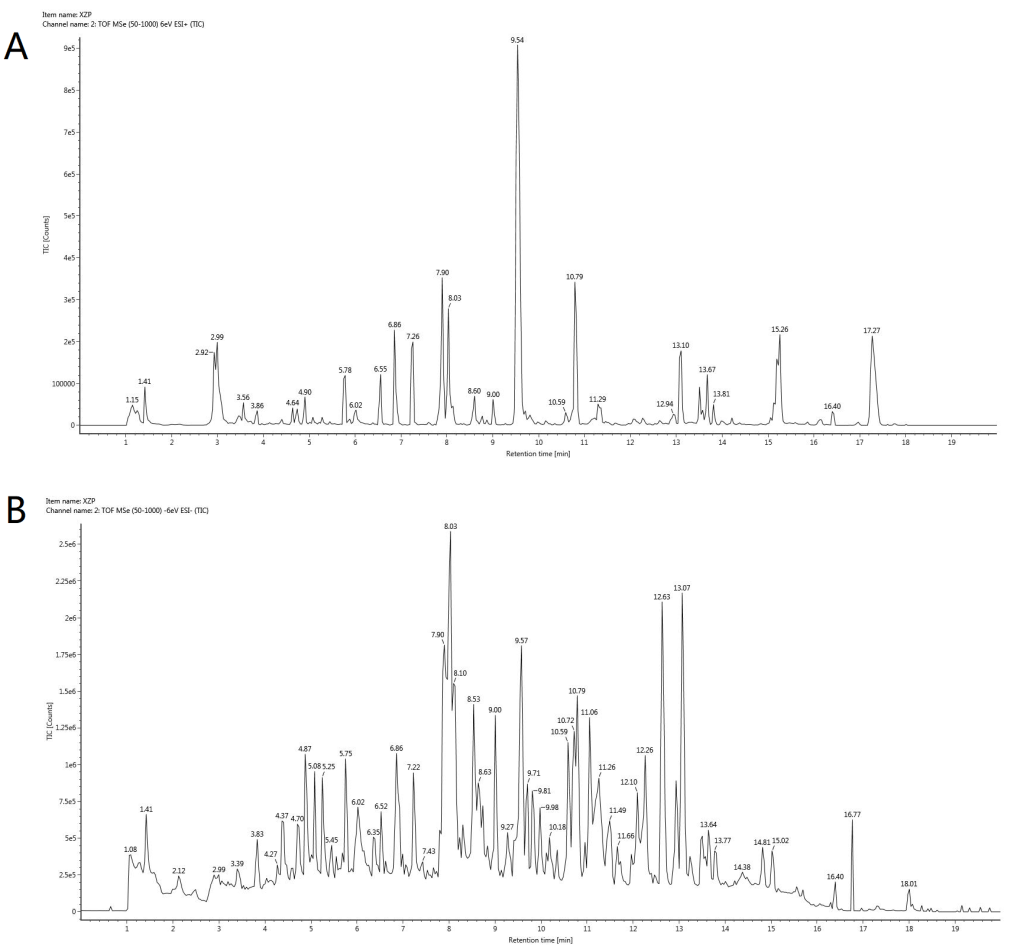


**Supplementary figure 1**：Liquid chromatography-mass spectrometry chromatograms for the extracts of XZP in **(A)** ESI+ and **(B)** ESI-.


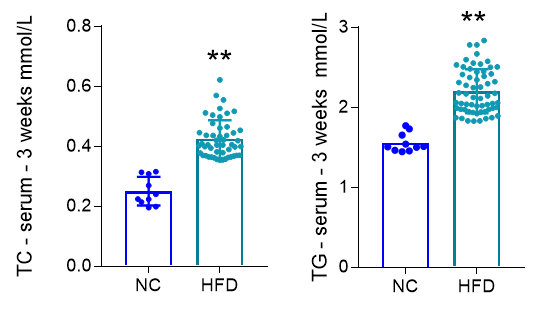


**Supplementary figure 2**：Serum TC and TG contents of hamsters induced by high-fat diet for 3 weeks


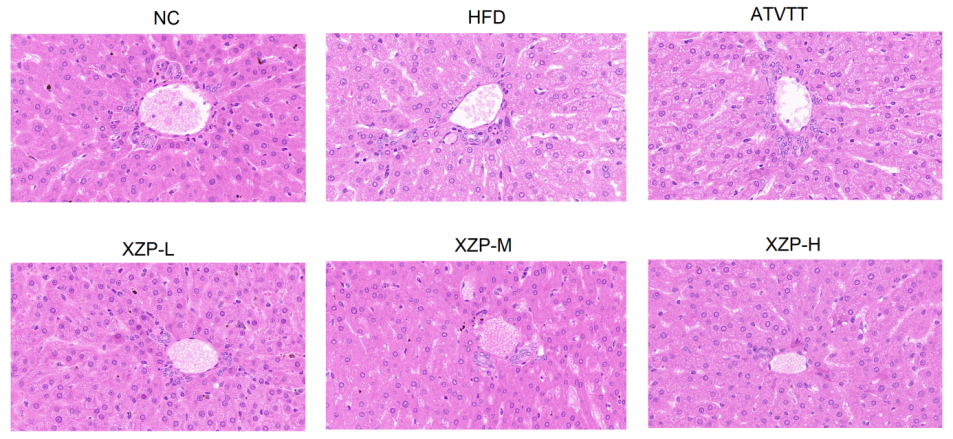


**Supplementary figure 3：**Serum TC and TG contents of hamsters induced by high-fat diet for 3 weeks HE staining of livers. (Scale bar = 100 μm)

# Supplementary Tables

**Supplementary table 1: Lipid, Liver function, Oxidative stress and Inflammation index of hamsters**

| **Indicator Type** | **Indicator** | **Sample Type** | **NC** | **HFD** | **ATVTT** | **XZP-L** | **XZP-M** | **XZP-H** |
| --- | --- | --- | --- | --- | --- | --- | --- | --- |
| Lipid expression index | TC | Serum | 3.59±0.38 | 4.28±0.39** | 3.48±0.49## | 3.18±0.74## | 3.22±0.49## | 3.46±0.33## |
|  |  | Liver | 6.70±1.14 | 18.39±2.32** | 8.89±2.40## | 13.29±4.43## | 15.11±3.34# | 14.01±5.25# |
|  | TG | Serum | 0.44±0.08 | 1.80±0.4** | 1.00±0.15## | 0.73±0.37## | 0.69±0.22## | 0.91±0.36*##* |
|  |  | Liver | 5.11±1.16 | 16.68±6.03** | 9.11±2.87## | 9.92±3.28## | 11.70±2.95# | 11.45±2.98# |
|  | HDL-C | Serum | 2.08±0.04 | 2.05±0.03 | 2.06±0.08 | 2.05±0.08 | 2.01±0.11 | 2.04±0.07 |
|  |  | Liver | 5.03±0.19 | 4.44±0.07** | 4.76±0.19## | 4.76±0.15## | 4.64±0.16## | 4.86±0.16## |
|  | LDL-C | Serum | 14.04±0.94 | 15.01±0.82* | 13.7±0.61## | 13.43±0.68## | 13.47±0.32## | 13.67±0.37## |
|  |  | Liver | 18.49±1.2 | 19.68±1.22* | 18.40±1.10# | 17.87±0.21## | 17.57±0.26## | 18.56±0.64# |
| Liver function index | GOT | Serum | 2.1±0.35 | 2.01±0.63 | 2±0.44 | 1.83±0.27 | 2.27±0.37 | 1.96±0.39 |
|  |  | Liver | 76.39±3.26 | 81.62±2.14** | 82.18±1.86 | 74.67±2.62## | 74.41±2.10## | 74.9±2.22## |
|  | GPT | Serum | 1.91±0.37 | 1.92±0.54 | 1.61±0.61 | 2.14±0.86 | 2.19±0.98 | 2.07±0.87 |
|  |  | Liver | 124.3±1.93 | 124.72±1.65 | 122.66±3.43 | 124.41±7.07 | 125.77±7.17 | 124±7.19 |
|  | ALP | Serum | 0.11±0.01 | 0.11±0.01 | 0.11±0.01 | 0.12±0.01 | 0.11±0.01 | 0.1±0.02 |
|  |  | Liver | 0.28±0.12 | 0.31±0.08 | 0.27±0.09 | 0.3±0.09 | 0.28±0.13 | 0.27±0.1 |
|  | GGT | Serum | 101.13±4.14 | 105.62±4.19* | 101.41±2.84# | 100.06±1.88## | 98.26±1.69## | 100.73±1.85## |
|  |  | Liver | 0.76±0.04 | 0.8±0.03* | 0.74±0.03## | 0.75±0.02## | 0.75±0.02## | 0.79±0.03 |
| Oxidative stress index | SOD | Serum | 69.54±10.47 | 57.84±7.19** | 65.45±9.37 | 70.23±6.05## | 67.53±12.42# | 64.08±13.56 |
|  |  | Liver | 163.67±23.32 | 127.18±31.73** | 144.16±21.11 | 201.56±25.14## | 190.96±36.46## | 141.89±15.12 |
|  | MDA | Serum | 11.36±2.38 | 11.84±1.57 | 13.09±4.01 | 13.52±3.84 | 13.93±3.17 | 9.94±3.07 |
|  |  | Liver | 9.22±4.72 | 9.22±1.48 | 8.93±3.71 | 8.44±3.41 | 9.66±2.34 | 10.92±2.84 |
|  | GSH | Serum | 120.22±19.34 | 123.05±17.76 | 127.95±15.95 | 126.38±30.51 | 109.5±14.38 | 108.33±16.89 |
|  |  | Liver | 2.22±0.20 | 1.98±0.16** | 2.11±0.31 | 2.24±0.23## | 2.37±0.21## | 2.57±0.14## |
| Inflammation index | IL-6 | Serum | 126.09±12.15 | 124.58±8.99 | 129.14±14.1 | 120.59±9.23 | 119.64±5.43 | 131.49±6.53 |
|  | GRP | Serum | 244.58±6.86 | 272.2±5.53** | 245.11±11.59## | 241.53±12.81## | 247.97±7.85## | 262.97±5.69## |

**Supplementary table 2: Fragments information of serum lipid metabolites**

| **No.** | **Compound Name** | **m/z** | **RT (min)** | **Adducts** | **Formula** | **Mass Error (ppm)** | **Mass fragments** |
| --- | --- | --- | --- | --- | --- | --- | --- |
| 1 | Prostaglandin E2 | 353.2318 | 6.14 | M+H | C20H32O5 | -1.29 | 353.2323 [C20H32O5+H], 335.2217 [C20H31O4-e], 317.2112 [C20H29O3-e], 233.1537 [C15H22O2-e], 95.0856 [C7H12-e] |
| 2 | Cholic acid | 453.2849 | 6.45 | M+FA-H | C24H40O5 | -2.18 | 453.2858 [C24H40O5+FA-H],423.2752 [C23H38O4+FA-H], 407.2803 [C23H38O3+FA-H], 405.2646 [C23H36O3+FA-H]，389.2697 [C24H38O4+e] |
| 3 | 11b-Hydroxyprogesterone | 355.1873 | 6.78 | M+Na | C20H28O4 | -1.97 | 333.2061 [C20H28O4+H], 315.1955 [C20H27O3-e], 297.1850 [C20H25O2-e], 273.1849 [C18H24O2+H], 189.1274 [C13H16O+H] |
| 4 | Alpha-Linolenic acid | 323.2220 | 7.02 | M+FA-H | C18H30O2 | -2.93 | 323.2228 [C18H30O2+FA-H], 305.2122[C18H28O+FA-H], 279.2329 [C18H30O2+e], 261.2223 [C18H29O+e] |
| 5 | Tetrahydrocortisol | 367.2471 | 7.05 | M+H | C21H34O5 | -2.10 | 367.2484 [C21H34O5+H], 349.2379 [C21H33O4+H], 331.2273 [C21H31O3+H], 289.2168 [C19H29O2+H] |
| 6 | 5,6-DHET | 337.2377 | 7.05 | M-H | C20H34O4 | -2.27 | 337.2384 [C20H33O4-H], 319.2279[C20H31O3-H], 293.2486 [C19H33O2-H], 275.2380[C19H31O-H] |
| 7 | 20-Hydroxyeicosatetraenoic acid | 319.2270 | 7.06 | M-H | C20H32O3 | -2.84 | 319.2273 [C20H32O3-H], 301.2168[C20H29O2-H], 289.2168 [C19H27O2-H], 275.2375[C19H37O-H] |
| 8 | Linoleic acid | 325.2375 | 7.14 | M+FA-H | C18H32O2 | -3.22 | 325.2384 [C18H32O2+FA-H], 307.2279 [C18H30O+FA-H], 281.2486 [C18H32O2+e], 263.2380 [C18H31O+e] |
| 9 | LysoPC(15:0) | 480.3081 | 7.30 | M-H | C23H48NO7P | -3.04 | 480.3096[C23H48NO7P-H], 255.2422[C16H31O2-H], 241.2173[C6H15NO4P-H] |
| 10 | LysoPC(16:0) | 540.3292 | 7.30 | M+FA-H | C24H50NO7P | -3.10 | 540.3307 [C24H50NO7P+FA-H], 480.3095 [C23H47NO7P+e], 255.2329 [C16H31O2+e], 242.0799 [C6H15NO4P+FA-H] |
| 11 | SM(d18:1/23:0) | 845.6729 | 7.33 | M+FA-H | C46H93N2O6P | -2.97 | 845.6753 [C46H93N2O6P+FA-H], 785.6542 [C45H90N2O6P+e], 354.3503 [C22H45+FA-H], 253.2173 [C15H29+FA-H], 242.0799 [C6H15NO4P+FA-H] |
| 12 | LysoPC(18:1(9Z)) | 566.3442 | 7.47 | M+FA-H | C26H52NO7P | -4.20 | 566.3463 [C26H52NO7P+FA-H], 506.3252 [C25H49NO7P+e], 281.2486 [C18H33O2+e], 242.0799 [C6H15NO4P+FA-H] |
| 13 | PE(18:0/20:1(11Z)) | 818.5894 | 7.50 | M+FA-H | C43H84NO8P | -2.97 | 818.5917 [C43H84NO8P+FA-H], 506.3252 [C25H49NO7P+e], 281.2486 [C17H33+FA-H], 183.0064 [C3H6O4P+FA-H], 59.0139 [CH2+FA-H] |
| 14 | LysoPC(17:0) | 508.3391 | 7.64 | M-H | C25H52NO7P | -3.41 | 494.3252 [C24H49NO7P+e], 283.2642 [C18H35O2+e], 269.2486 [C17H33O2+e] |
| 15 | LysoPC(18:0) | 568.3603 | 7.64 | M+FA-H | C26H54NO7P | -3.27 | 568.3620 [C26H54NO7P+FA-H], 508.3408 [C25H51NO7P+e], 283.2642[C18H35O2+e], 255.2330 [C15H31+FA-H], 242.0799 [C6H15NO4P+FA-H] |
| 16 | LysoPC(20:1(11Z)) | 594.3762 | 7.84 | M+FA-H | C28H56NO7P | -2.55 | 594.3776 [C28H56NO7P+FA-H], 534.3565 [C27H53NO7P+e], 309.2799[C20H37O2+e], 242.0799 [C6H15NO4P+FA-H] |
| 17 | LysoPC(22:1(13Z)) | 622.4079 | 8.28 | M+FA-H | C30H60NO7P | -1.81 | 622.4089 [C30H60NO7P+FA-H], 562.3878 [C29H57NO7P+e], 337.3112 [C22H41O2+e] |
| 18 | SM(d18:1/12:0) | 691.5018 | 8.77 | M+FA-H | C35H71N2O6P | -2.08 | 691.5032 [C35H71N2O6P+FA-H], 631.4820 [C34H68N2O6P+e] |
| 19 | Galabiosylceramide (d18:1/16:0) | 906.6143 | 9.48 | M+FA-H | C46H87NO13 | -1.95 | 860.6104 [C46H87NO13+e], 769.5709 [C43H79NO10+e], 536.5048 [C34H66NO3+e], 221.0666 [C8H14O7+e], 179.0561 [C6H11O6+e] |
| 20 | SM(d18:0/24:1(15Z)) | 859.6892 | 9.68 | M+FA-H | C47H95N2O6P | -2.17 | 859.6910 [C47H95N2O6P+FA-H], 799.6698 [C46H92N2O6P+e], 283.2643 [C17H33+FA-H], 281.2486 [C17H33+FA-H] |
| 21 | PC(16:0/22:6(4Z,7Z,10Z,13Z,16Z,19Z)) | 850.5579 | 10.30 | M+FA-H | C46H80NO8P | -3.05 | 850.5604 [C46H80NO8P+FA-H], 790.5392 [C45H77NO8P+e], 329.2486 [C21H31+FA-H], 283.2431 [C21H31+e], 255.2329 [C16H31O2+e] |
| 22 | PC(14:0/P-18:0) | 718.5730 | 10.92 | M+H | C40H80NO7P | -2.18 | 718.5745 [C40H80NO7P+H], 632.4650 [C34H67NO7P-e], 184.0733 [C5H13NO4P+H], 104.1070 [C5H13NO+H], 86.0965 [C5H13N-e] |
| 23 | PC(16:0/P-18:1(11Z)) | 744.5883 | 11.09 | M+H | C42H82NO7P | -2.50 | 744.5902 [C42H82NO7P+H], 561.5242 [C37H69O3-e], 506.3605 [C26H51NO6P+H], 184.0733 [C5H13NO4P+H], 104.1070 [C5H13NO+H] |
| 24 | PC(18:0/22:6(4Z,7Z,10Z,13Z,16Z,19Z)) | 878.5893 | 11.27 | M+FA-H | C48H84NO8P | -2.81 | 878.5917 [C48H84NO8P+FA-H], 818.5705 [C47H81NO8P+e], 329.2486 [C21H31+FA-H], 255.2330 [C15H31+FA-H], 229.1961 [C17H25+e] |
| 25 | Glucosylceramide (d18:1/20:0) | 800.6238 | 11.46 | M+FA-H | C44H85NO8 | -2.48 | 800.6257 [C44H85NO8+FA-H], 754.6202 [C44H85NO8+e], 592.5674 [C38H74NO3+e], 336.3271 [C22H43NO+e], 119.0350 [C3H6O2+FA-H] |
| 26 | PC(18:1(9Z)/18:1(9Z)) | 786.5987 | 11.51 | M+H | C44H84NO8P | -2.63 | 786.6008 [C44H84NO8P+H], 603.5347 [C39H71O4-e], 522.3554 [C26H51NO7P+H], 506.3605 [C26H51NO6P+H], 166.0628 [C5H13NO3P-e] |
| 27 | PC(16:0/P-18:0) | 746.6038 | 11.81 | M+H | C42H84NO7P | -2.75 | 746.6058 [C42H84NO7P+H], 563.5398 [C37H71O3-e], 508.3762 [C26H53NO6P+H], 166.0628 [C5H13NO3P-e], 104.1070 [C5H13NO+H] |
| 28 | PC(14:0/20:0) | 762.5987 | 11.98 | M+H | C42H84NO8P | -2.63 | 784.5827 [C42H84NO8P+Na], 579.5347 [C37H71O4-e], 166.0628 [C5H13NO3P-e], 104.1070 [C5H13NO-e], 86.0965 [C5H13N-e] |
| 29 | Galabiosylceramide (d18:1/24:1(15Z)) | 1016.7239 | 12.18 | M+FA-H | C54H101NO13 | -1.60 | 970.7200 [C54H101NO13+e], 253.0928 [C9H16O8+e] |
| 30 | PE(18:1(11Z)/24:1(15Z)) | 850.6295 | 12.43 | M+Na | C47H90NO8P | -0.15 | 828.6477 [C47H90NO8P+H], 827.6399 [C47H90NO8P-e], 811.6212 [C47H88O8P-e], 71.0856 [C5H11-e] |
| 31 | Glucosylceramide (d18:1/22:0) | 783.6559 | 12.65 | M-H | C46H89NO8 | -3.72 | 782.6515 [C46H89NO8-H], 710.6148[C44H86O6-H], 620.5987 [C44H44O3-H] |
| 32 | SM(d18:0/22:1(13Z)) | 831.6582 | 13.22 | M+FA-H | C45H91N2O6P | -1.90 | 831.6597 [C45H91N2O6P+FA-H], 771.6385 [C44H88N2O6P+e], 770.5943[C42H82N2O5P+FA-H], 281.2486 [C17H33+FA-H], 255.2330 [C15H31+FA-H] |
| 33 | PC(14:0/22:0) | 790.6301 | 13.28 | M+H | C44H88NO8P | -2.41 | 812.6140 [C44H88NO8P+Na], 104.1070 [C5H13NO-e], 86.0965 [C5H13N-e], 58.0652 [C3H9N-e], 57.0699 [C4H9-e] |
| 34 | PC(14:0/24:1(15Z)) | 816.6456 | 13.54 | M+H | C46H90NO8P | -2.56 | 816.6333[C46H90NO8P+H],798.6371[C44H875NO7P+H], 798.6371[C41H75NO4+H],349.3465 [C24H45O+H],211.2056 [C14H27O+H] |

**Supplementary table 3: Intensity of serum metabolites**

| **Metabolite type** | **Compound Name** | **NC/ Intensity** | **HFD/ Intensity** | **XZP-L/ Intensity** | **XZP-M/ Intensity** | **XZP-H/ Intensity** |
| --- | --- | --- | --- | --- | --- | --- |
| PC | PC(18:1(9Z)/18:1(9Z)) | 493427882±118851780 | 263463199±111806229** | 363099885±48154932# | 353711332±74303928# | 362463972±87528890# |
|  | PC(14:0/P-18:0) | 19500851±4484131 | 12663538±3404552** | 17540087±5274557# | 18799054±4919500## | 13635700±4572637 |
|  | PC(16:0/P-18:1(11Z)) | 8052055±2754498 | 5342691±1868143* | 7366584±1756962# | 8390057±2555007## | 6166161±2018477 |
|  | PC(16:0/P-18:0) | 46922720±11326122 | 26000527±5885467** | 35546000±8123892## | 39643552±12190663## | 29741054±8684118 |
|  | PC(18:0/22:6(4Z,7Z,10Z,13Z,16Z,19Z)) | 8852362±1390045 | 20283963±3104066** | 17155105±2742280# | 14491227±3062968## | 19600696±3644056 |
|  | PC(14:0/20:0) | 21304275±5258175 | 30717169±6038602** | 25287030±5496058# | 23893941±5509576# | 29989747±7138212 |
|  | PC(14:0/22:0) | 3079380±867043 | 5519333±1618227** | 3252199±680137## | 3606445±1514189# | 4811865±1928821 |
|  | PC(14:0/24:1(15Z)) | 6098363±1370566 | 10855161±2712529** | 7902069±2780796# | 6668657±1619153## | 10236094±3469499 |
|  | PC(16:0/22:6(4Z,7Z,10Z,13Z,16Z,19Z)) | 49602126±6220368 | 58607169±6136658** | 51553400±5566015# | 43579842±8795533## | 55668264±10147867 |
| SM | SM(d18:0/22:1(13Z)) | 7841741±673023 | 14506057±2842855** | 12085530±1249864# | 11801896±2308442# | 12222058±869582# |
|  | SM(d18:1/23:0) | 255158±43953 | 502676±156553** | 344220±110121# | 322028±160533# | 356884±122193# |
|  | SM(d18:1/12:0) | 48377±17023 | 82778±17901** | 60575±21483# | 61558±19139# | 49956±6512## |
|  | SM(d18:0/24:1(15Z)) | 6511471±993748 | 14240436±3186524** | 11257871±2077623# | 13988827±3218388 | 10377540±2004515## |
| PE | PE(18:1(11Z)/24:1(15Z)) | 2576318±474897 | 5562184±1966644** | 3480295±926462## | 3283033±1078058## | 4882889±1529782 |
|  | PE(18:0/20:1(11Z)) | 34092±24234 | 63596±19337** | 39490±12678## | 43914±13344# | 37236±17287## |
| Cer | Glucosylceramide (d18:1/20:0) | 25932±11271 | 178703±52445** | 124204±46605# | 172103±64720 | 113947±22658## |
|  | Glucosylceramide (d18:1/22:0) | 1157381±292326 | 5073315±2390554** | 3182095±1190639# | 4106873±2367403 | 2791995±678440## |
|  | Galabiosylceramide (d18:1/24:1(15Z)) | 24086±12452 | 88025±32300** | 60035±4636# | 59607±19628# | 45743±17084## |
|  | Galabiosylceramide (d18:1/16:0) | 240696±56099 | 357246±32475** | 287908±94989# | 264557±107184# | 290642±76891# |
| LysoPC | LysoPC(18:1(9Z)) | 86368836±7404884 | 98281022±8056288** | 87928248±4612158## | 86890754±8474784## | 88372137±7569642# |
|  | LysoPC(17:0) | 913196±103303 | 1405933±313730** | 1151293±196489# | 1120974±153190# | 1306820±187296 |
|  | LysoPC(18:0) | 35372070±4018048 | 49068384±4552008** | 39612861±8555184## | 41814221±5400769## | 46911064±6445174 |
|  | LysoPC(20:1(11Z)) | 2244644±349097 | 2754789±382583** | 2200554±637409# | 2264856±574992# | 3078324±595875 |
|  | LysoPC(16:0) | 38297907±3060376 | 45031019±3247800** | 33831675±14907740# | 41119616±4495769# | 44675918±4642272 |
|  | LysoPC(22:1(13Z)) | 249424±33095 | 304844±44337** | 232131±80700# | 239251±55748## | 308517±90466 |
|  | LysoPC(15:0) | 1268901±111040 | 1674179±150294** | 1356557±407770# | 1497734±163842# | 1617265±168761 |
| Other metabolites | 5,6-DHET | 79141±23376 | 270480±64699** | 206371±47313# | 210303±40781# | 216727±29107# |
|  | 20-Hydroxyeicosatetraenoic acid | 1125453±460448 | 2437975±610571** | 1761638±560186# | 1076428±901960## | 1810631±858492 |
|  | Tetrahydrocortisol | 11880±4649 | 159413±42013** | 151214±42675 | 148630±41878 | 126228±22484# |
|  | Linoleic acid | 91759±32907 | 222419±40738** | 182320±39195# | 181337±36192# | 184662±28940# |
|  | Cholic acid | 343470±151851 | 49985±21823** | 450647±158124## | 336206±94779## | 558196±292145## |
|  | Prostaglandin E2 | 97138±25318 | 39654±6024** | 53742±18243# | 50637±14269# | 69486±22553## |
|  | Alpha-Linolenic acid | 244505±45050 | 77903±10757** | 277136±54985## | 256325±33740## | 240768±33256## |
|  | 11b-Hydroxyprogesterone | 194580±54541 | 33396±13282** | 215554±62135## | 234647±37375## | 226585±53582## |

**Supplementary table 4: Fragments information of liver lipid metabolites**

| **No** | **Compound Name** | **m/z** | **RT (min)** | **Adducts** | **Formula** | **Mass Error (ppm)** | **Mass fragments** |
| --- | --- | --- | --- | --- | --- | --- | --- |
| 1 | Taurocholic acid | 1029.5738 | 6.56 | 2M-H | C26H45NO7S | -2.24 | 1029.5761[C26H45NO7S-H+M], 514.2844 [C26H45NO7S-H], 496.2738 [C26H43NO6S-H], 371.2591 [C24H37O3-H], 106.9808 [C2H5O3S-H] |
| 2 | Glycocholic acid | 929.6086 | 6.60 | 2M-H | C26H43NO6 | -2.40 | 929.6108 [C26H43NO6-H+M], 464.3017 [C26H43NO6-H], 402.3013 [C25H40NO3+e], 400.2857 [C25H40NO3-H] |
| 3 | Lithocholic acid glycine conjugate | 478.3159 | 6.66 | M+FA-H | C26H43NO4 | -3.58 | 478.3174 [C26H43NO4+FA-H],446.2912 [C25H39NO3+FA-H] |
| 4 | Bilirubin glucuronide | 759.2869 | 6.66 | M-H | C39H44N4O12 | -1.87 | 759.2883 [C39H44N4O12-H], 285.1244 [C16H17N2O3+e], 113.0244 [C5H6O3-H], 85.0295 [C4H5O2+e], 59.0138 [C2H3O2+e] |
| 5 | Alpha-Linolenic acid | 279.2315 | 6.75 | M+H | C18H30O2 | -1.45 | 279.2319 [C18H30O2+H], 243.2108 [C18H28-e], 209.1537 [C13H21O2-e], 163.1482 [C12H19-e], 151.1482 [C11H17+H] |
| 6 | Sphingosine | 300.2892 | 6.75 | M+H | C18H37NO2 | -1.70 | 300.2897 [C18H37NO2+H], 283.2632 [C18H35O2-e], 282.2792 [C18H36NO-e], 239.2370 [C16H31O-e], 99.1169 [C7H15-e] |
| 7 | Deoxycholic acid | 437.2896 | 6.76 | M-H | C24H40O4 | -3.27 | 391.2854 [C24H40O4-H], 345.2799 [C23H38O2-H], 327.2693 [C23H37O-H], 110.0737 [C7H10O+e], 89.0244 [C2H4O+FA-H] |
| 8 | 5b-Cyprinol sulfate | 531.2987 | 6.77 | M-H | C27H48O8S | -1.89 | 531.2997 [C27H84O8S-H], 530.2919 [C27H48O8S-H], 513.2891 [C27H46O7S-H], 95.9523 [HO4S-H], 79.9573 [HO3S-H] |
| 9 | LysoPC(18:4(6Z,9Z,12Z,15Z)) | 560.2984 | 6.96 | M+FA-H | C26H46NO7P | -1.97 | 500.2782 [C25H43NO7P+e], 275.2016 [C18H27O2+e] |
| 10 | LysoPC(18:3(6Z,9Z,12Z)) | 562.3136 | 7.04 | M+FA-H | C26H48NO7P | -2.87 | 562.3150 [C26H48NO7P+FA-H], 502.2939 [C25H45NO7P+e], 242.0799 [C6H15NO4P+FA-H], 233.2274 [C17H29+e],127.0765 [C6H10+FA-H] |
| 11 | D-Urobilinogen | 591.3168 | 7.06 | M+H | C33H42N4O6 | -1.61 | 575.2870[C33H42N4O6+H],466.2337 [C26H32N3O5-e] , 301.1547 [C17H21N2O3-e] , 273.1598 [C16H19N2O2+H] , 138.0914 [C8H12NO-e] , 124.0757 [C7H10NO-e] |
| 12 | LysoPC(20:4(8Z,11Z,14Z,17Z)) | 566.3229 | 7.09 | M+Na | C28H50NO7P | 2.24 | 566.3242[C28H50NO7P+Na],361.2738 [C23H37O3-e], 258.1101 [C8H19NO6P+H], 203.1795 [C15H23-e]， 184.0733 [C5H13NO4P+H], 166.0628 [C5H13NO3P-e] |
| 13 | LysoPC(22:6(4Z,7Z,10Z,13Z,16Z,19Z)) | 612.3290 | 7.14 | M+FA-H | C30H50NO7P | -2.95 | 612.3307 [C30H50NO7P+FA-H], 552.3095 [C29H47NO7P+e], 327.2329 [C22H31O2+e], 283.2431 [C21H31+e], 242.0799 [C6H15NO4P+FA-H] |
| 14 | LysoPC(20:4(5Z,8Z,11Z,14Z)) | 544.3387 | 7.24 | M+H | C28H50NO7P | -1.98 | 544.3398 [C28H50NO7P+H], 526.3292 [C28H49NO6P-e], 184.0733 [C5H13NO4P+H], 104.1070 [C5H13NO+H], 60.0808 [C3H9N+H] |
| 15 | LysoPC(22:5(4Z,7Z,10Z,13Z,16Z)) | 570.3541 | 7.33 | M+H | C30H52NO7P | -2.30 | 570.3554 [C30H52NO7P+H], 552.3449 [C30H51NO6P-e], 387.2894 [C25H39O3-e], 184.0733 [C5H13NO4P+H], 104.1070 [C5H13NO+H] |
| 16 | LysoPC(22:5(7Z,10Z,13Z,16Z,19Z)) | 614.3451 | 7.41 | M+FA-H | C30H52NO7P | -2.14 | 614.3463 [C30H52NO7P+FA-H], 554.3252 [C29H49NO7P+e], 329.2486 [C22H33O2+e], 285.2587 [C21H33+e], 59.0139 [CH3+FA-H] |
| 17 | PE(18:3(6Z,9Z,12Z)/P-18:0) | 770.5329 | 7.42 | M+FA-H | C41H76NO7P | -1.73 | 770.5341 [C41H76NO7P+FA-H], 480.3096 [C22H45NO5P+FA-H], 279.2330 [C17H29+DA-H], 255.2330 [C15H31+FA-H], 152.9958 [C2H4O3P+FA-H] |
| 18 | LysoPC(20:2(11Z,14Z)) | 570.3541 | 7.42 | M+Na | C28H54NO7P | 1.99 | 570.3541[C28H54NO7P+Na]530.3606 [C28H53NO6P-e], 365.3051 [C23H41O3-e], 258.1101 [C8H19NO6P+H], 184.0733 [C5H13NO4P+H], 166.0628 [C5H13NO3P-e] |
| 19 | LysoPC(22:4(7Z,10Z,13Z,16Z)) | 616.3604 | 7.51 | M+FA-H | C30H54NO7P | -2.75 | 616.3620 [C30H54NO7P+FA-H], 556.3408 [C29H51NO7P+e], 331.2642 [C22H35O2+e], 287.2744 [C21H35+e], 242.0799 [C6H15NO4P+FA-H] |
| 20 | LysoPC(16:0) | 494.3242 | 7.56 | M-H | C24H50NO7P | -2.01 | 494.3252 [C24H50NO7P-H], 279.2329 [C18H33O2-H], 267.2329 [C17H33O2-H], 255.2329 [C16H31O2+e], 224.0693 [C7H17NO5P-H] |
| 21 | LysoPC(20:1(11Z)) | 550.3858 | 7.82 | M+H | C28H56NO7P | -1.75 | 550.3867 [C28H56NO7P+H], 549.3789 [C28H56NO7P-e], 367.3207 [C23H43O3-e], 184.0733 [C5H13NO4P+H], 104.1070 [C5H13NO+H] |
| 22 | 12,13-DHOME | 313.2373 | 8.00 | M-H | C18H34O4 | -3.47 | 313.2384 [C18H34O4-H], 279.2329 [C18H31O2+e], 255.2329 [C16H31O2+e], 59.0138 [C2H3O2+e] |
| 23 | 8,11,14-Eicosatrienoic acid | 305.2476 | 8.08 | M-H | C20H34O2 | -3.39 | 304.2408 [C20H34O2-H], 72.0217 [C3H5O2-H], 59.0138 [C2H3O2+e] |
| 24 | LysoPC(20:0/0:0) | 552.4018 | 8.19 | M+H | C28H58NO7P | -0.94 | 552.4011[C28H58NO7P+H]240.0996 [C8H19NO5P-e], 166.0628 [C5H13NO3P-e], 104.1070 [C5H13NO+H], 86.0965 [C5H13N-e] |
| 25 | PS(18:0/18:1(9Z)) | 790.5579 | 8.57 | M+H | C42H80NO10P | -1.77 | 790.5593 [C42H80NO10P+H], 772.5487 [C42H79NO9P-e], 756.5538 [C42H78NO8P+H], 754.5382 [C42H77NO8P-e], 124.9998 [C2H5O4P+H] |
| 26 | PI(18:0/18:2(9Z,12Z)) | 861.5481 | 8.71 | M-H | C45H83O13P | -2.04 | 861.5498 [C45H83O13P-H], 599.3201 [C27H52O12P+e], 577.2783 [C27H46O11P+e], 283.2642 [C18H35O2+e], 281.2486 [C18H35O2-H] |
| 27 | 25-Hydroxycholesterol | 403.3570 | 8.78 | M+H | C27H46O2 | -0.23 | 403.3566[C27H46O2+H]385.3465 [C27H45O-e] , 367.3360 [C27H43-e] , 159.1169 [C12H16-e] , 145.1012 [C11H14-e] , 107.0856 [C8H10+H] |
| 28 | Cer(d18:1/24:1(15Z)) | 692.6185 | 8.81 | M+FA-H | C42H81NO3 | -2.04 | 692.6198 [C42H81NO3+FA-H], 646.6143 [C42H81NO3+e], 390.3741 [C26H48NO+e] |
| 29 | PE(14:0/22:5(7Z,10Z,13Z,16Z,19Z)) | 738.5047 | 8.83 | M+H | C41H72NO8P | -2.93 | 738.5069 [C41H72NO8P+H], 597.4878 [C39H65O4-e], 124.9999 [C2H5O4P+H], 95.0856 [C7H11-e], 81.0699 [C6H10-e] |
| 30 | Bilirubin | 585.2691 | 9.02 | M+H | C33H36N4O6 | -2.89 | 584.2630 [C33H36N4O6-e] , 299.1391 [C17H19N2O3-e] , 286.1312 [C16H17N2O3+H] , 284.1156 [C16H17N2O3-e] , 255.1129 [C15H15N2O2-e] |
| 31 | PIP2(16:0/16:1(9Z)) | 967.4392 | 9.62 | M-H | C41H79O19P3 | 3.75 | 966.4277 [C41H79O19P3-H], 948.4172 [C41H77O18P3-H], 150.9802 [C3H5O5P-H], 120.9696 [C2H3O4P-H] |
| 32 | 24-Methylenecholesterol | 399.3614 | 9.68 | M+H | C28H46O | -1.84 | 399.3622 [C28H46O+H], 367.3360 [C27H42+H], 159.1169 [C12H16-e], 145.1012 [C11H14-e], 137.0961 [C9H14O-e] |
| 33 | PC(16:1(9Z)/22:6(4Z,7Z,10Z,13Z,16Z,19Z)) | 848.5427 | 9.70 | M+FA-H | C46H78NO8P | -2.50 | 848.5447 [C46H78NO8P+FA-H], 788.5235 [C45H75NO8P+e], 327.2329 [C22H31O2+e], 283.2431 [C21H31+e], 255.2330 [C15H29+FA-H] |
| 34 | PE(16:0/18:3(9Z,12Z,15Z)) | 714.5061 | 9.87 | M+H | C39H72NO8P | -1.05 | 714.5069 [C39H72NO8P+H], 137.1325 [C10H15+H], 124.9998 [C2H5O4P+H], 123.1169 [C9H14+H], 83.0856 [C6H10+H] |
| 35 | PE(16:0/22:5(7Z,10Z,13Z,16Z,19Z)) | 788.5197 | 9.94 | M+Na | C43H76NO8P | -0.53 | 788.5201 [C43H76NO8P+Na], 647.5010 [C41H69O4+Na], 124.9999 [C2H5O4P-e], 105.0675 [C6H10+Na], 91.0518 [C5H9+Na] |
| 36 | PC(18:2(9Z,12Z)/22:6(4Z,7Z,10Z,13Z,16Z,19Z)) | 874.5575 | 10.01 | M+FA-H | C48H80NO8P | -3.43 | 874.5604 [C48H80NO8P+FA-H], 814.5392 [C47H77NO8P+e], 329.2486 [C21H31+FA-H], 281.2486 [C17H31+FA-H], 253.2173 [C15H27+FA-H] |
| 37 | PC(16:0/20:4(5Z,8Z,11Z,14Z)) | 826.5569 | 10.19 | M+FA-H | C44H80NO8P | -4.44 | 826.5604 [C44H80NO8P+FA-H], 766.5392 [C43H77NO8P+e], 277.2173 [C17H27+FA-H], 255.2329 [C16H31O2+e], 205.1961 [C15H24+e] |
| 38 | PC(16:0/22:6(4Z,7Z,10Z,13Z,16Z,19Z)) | 850.5580 | 10.31 | M+FA-H | C46H80NO8P | -2.95 | 850.5604 [C46H80NO8P+FA-H], 790.5392 [C45H77NO8P+e], 327.2329 [C22H31O2+e], 283.2431 [C21H31+e], 255.2329 [C16H31O2+e] |
| 39 | PE(16:0/22:6(4Z,7Z,10Z,13Z,16Z,19Z)) | 764.5209 | 10.31 | M+H | C43H74NO8P | -2.02 | 764.5225 [C43H74NO8P+H], 625.5191 [C41H67O4+H], 623.5034 [C41H67O4-e], 203.1795 [C15H22+H], 97.1012 [C7H11+H] |
| 40 | PC(14:0/18:1(11Z)) | 776.5425 | 10.32 | M+FA-H | C40H78NO8P | -3.00 | 775.5369 [C40H78NO8P+FA-H], 564.4759 [C35H65O5+e], 255.2330 [C15H29+FA-H], 253.2173 [C15H29+FA-H] |
| 41 | PE(18:1(11Z)/20:4(5Z,8Z,11Z,14Z)) | 764.5217 | 10.70 | M-H | C43H76NO8P | -2.51 | 764.5236 [C43H76NO8P-H], 489.2623 [C24H43O8P-H], 304.2407 [C20H31O2+e], 279.2329 [C18H33O2-H], 140.0118 [C2H7NO4P+e] |
| 42 | PE(18:1(11Z)/18:2(9Z,12Z)) | 740.5221 | 10.77 | M-H | C41H76NO8P | -2.01 | 739.5157 [C41H76NO8P-H], 478.2939 [C23H45NO7P+e], 277.2173 [C18H31O2-H], 122.0012 [C2H7NO3P-H] |
| 43 | PE(20:0/22:6(4Z,7Z,10Z,13Z,16Z,19Z)) | 864.5733 | 10.83 | M+FA-H | C47H82NO8P | -3.27 | 864.5760 [C47H82NO8P+FA-H], 804.5548 [C46H79NO8P+e], 463.2255 [C25H37O6P+e], 329.2486 [C21H31+FA-H], 269.2486 [C16H33+FA-H] |
| 44 | PE(18:0/20:2(11Z,14Z)) | 816.5743 | 10.95 | M+FA-H | C43H82NO8P | -2.24 | 816.5760 [C43H82NO8P+FA-H], 756.5548 [C42H79NO8P+e], 489.2623 [C23H41O6P+FA-H], 327.2541 [C18H35O2+FA-H], 281.2486 [C17H31+FA-H] |
| 45 | PE(18:0/22:4(7Z,10Z,13Z,16Z)) | 840.5738 | 10.97 | M+FA-H | C45H82NO8P | -2.71 | 840.5760 [C45H82NO8P+FA-H], 780.5548 [C44H79NO8P+e], 305.2486 [C19H31+FA-H], 269.2486 [C16H33+FA-H], 205.1961 [C15H24+e] |
| 46 | PE(18:2(9Z,12Z)/P-18:0) | 750.5421 | 11.00 | M+Na | C41H78NO7P | 1.71 | 750.5408 [C41H78NO7P+Na], 609.5217 [C39H71O3+Na], 124.9999 [C2H5O4P-e], 121.0988 [C7H15+Na], 109.0988 [C6H13+Na] |
| 47 | PE(18:0/22:6(4Z,7Z,10Z,13Z,16Z,19Z)) | 792.5521 | 11.21 | M+H | C45H78NO8P | -2.16 | 792.5538 [C45H78NO8P+H], 651.5347 [C43H71O4-e], 652.5425 [C43H71O4+H], 267.2683 [C18H35O-e] |
| 48 | PE(18:0/P-18:1(11Z)) | 752.5576 | 11.23 | M+Na | C41H80NO7P | 1.57 | 611.5373 [C39H73O3+Na], 93.0675 [C5H11+Na], 81.0675 [C4H9+Na], 57.0699 [C4H9-e] |
| 49 | PE(18:0/18:2(9Z,12Z)) | 744.5527 | 11.46 | M+H | C41H78NO8P | -1.46 | 744.5538 [C41H78NO8P+H], 604.5425 [C39H71O4+H], 263.2370 [C18H31O-e], 137.1325 [C10H17-e], 124.9998 [C2H5O4P+H] |
| 50 | PC(18:0/20:3(5Z,8Z,11Z)) | 856.6050 | 11.90 | M+FA-H | C46H86NO8P | -2.84 | 856.6073 [C46H86NO8P+FA-H], 795.5783 [C45H83NO8P+e], 307.2643 [C19H33+FA-H], 283.2642 [C18H35O2+e], 279.2330 [C17H29+FA-H] |
| 51 | PC(16:0/22:5(7Z,10Z,13Z,16Z,19Z)) | 830.5673 | 12.05 | M+Na | C46H82NO8P | 0.34 | 830.5670 [C46H82NO8P+Na], 145.0988 [C9H14+Na], 131.0831 [C8H13+Na], 117.0675 [C7H11+Na] |
| 52 | PE(15:0/24:1(15Z)) | 832.6055 | 12.25 | M+FA-H | C44H86NO8P | -2.32 | 832.6073 [C44H86NO8P+FA-H], 772.5861 [C43H83NO8P+e], 391.2255 [C19H37O6P+e], 309.2799 [C19H37+FA-H], 283.2643 [C17H33+FA-H] |

**Supplementary table 5: Intensity of liver metabolites**

| **Metabolite type** | **Compound Name** | **NC/ Intensity** | **HFD/ Intensity** | **XZP-L/ Intensity** | **XZP-M/ Intensity** | **XZP-H/ Intensity** |
| --- | --- | --- | --- | --- | --- | --- |
| PC | PC(18:2(9Z,12Z)/22:6(4Z,7Z,10Z,13Z,16Z,19Z)) | 10617840±1254667 | 8117993±1148715** | 9772048±1557573# | 9176393±864587# | 9065053±788564# |
|  | PC(16:0/20:4(5Z,8Z,11Z,14Z)) | 25529206±1870799 | 8643226±738919** | 10171604±1989907# | 10303953±2034903# | 10199029±1403507## |
|  | PC(14:0/18:1(11Z)) | 4185502±807648 | 1234364±202356** | 1387439±249775 | 1555777±365191# | 1570986±269612## |
|  | PC(18:0/20:3(5Z,8Z,11Z)) | 8735983±856976 | 6261767±752863** | 7569499±1783842# | 8422446±1445916## | 9338906±1682270## |
|  | PC(16:0/22:5(7Z,10Z,13Z,16Z,19Z)) | 1034160±152416 | 420419±104616** | 546950±110358# | 539626±99569# | 561896±87787## |
|  | PC(16:1(9Z)/22:6(4Z,7Z,10Z,13Z,16Z,19Z)) | 17803844±1555527 | 5116788±584717** | 6303063±1023365## | 6378763±1517414# | 6950742±1155663## |
|  | PC(16:0/22:6(4Z,7Z,10Z,13Z,16Z,19Z)) | 63165888±1947065 | 51042507±2540434** | 57674764±6960154# | 57441536±5736084## | 58293064±4483951## |
| PE | PE(18:3(6Z,9Z,12Z)/P-18:0) | 100610±32718 | 424174±144974** | 249062±110932## | 215056±81542## | 202909±129753## |
|  | PE(16:0/22:6(4Z,7Z,10Z,13Z,16Z,19Z)) | 52264304±6254436 | 36542790±7169401** | 45016062±3621826## | 46582258±7554485## | 42823803±5177895# |
|  | PE(18:1(11Z)/20:4(5Z,8Z,11Z,14Z)) | 37836322±4057958 | 21107234±1425668** | 21710492±2560101 | 25594041±1323418## | 26694461±1986053## |
|  | PE(18:1(11Z)/18:2(9Z,12Z)) | 15647144±2207267 | 7520876±1310903** | 8969378±994468# | 9107516±616132## | 9519935±1614617## |
|  | PE(20:0/22:6(4Z,7Z,10Z,13Z,16Z,19Z)) | 1757491±176545 | 1325753±177173** | 1757945±396061## | 1644833±171843## | 1700591±178277## |
|  | PE(18:0/20:2(11Z,14Z)) | 7792878±495916 | 2054682±220786** | 2476751±355240## | 2483482±237643## | 2629121±234078## |
|  | PE(18:0/22:4(7Z,10Z,13Z,16Z)) | 4690196±264413 | 2206466±261083** | 2848636±585310## | 2981029±317927## | 3013208±205109## |
|  | PE(18:2(9Z,12Z)/P-18:0) | 2885618±481631 | 1969743±222433** | 2412177±347172## | 2563112±604583## | 2468404±225631## |
|  | PE(18:0/22:6(4Z,7Z,10Z,13Z,16Z,19Z)) | 28230871±5307439 | 23076111±3081270** | 28728462±4386490## | 28143243±3297693## | 29712130±3567360## |
|  | PE(18:0/P-18:1(11Z)) | 101982±25428 | 81909±15099** | 131670±57284# | 133187±43872## | 113855±31366## |
|  | PE(18:0/18:2(9Z,12Z)) | 17614960±2789646 | 11339156±547865** | 13379300±1367908## | 12611073±1154895## | 11965641±1892105 |
|  | PE(15:0/24:1(15Z)) | 14665792±1882389 | 8694140±753374** | 11387351±3309896# | 11214548±1146037## | 11098326±885109## |
|  | PE(14:0/22:5(7Z,10Z,13Z,16Z,19Z)) | 101200±40116 | 50826±23321** | 87925±37359# | 81323±36557# | 100791±52190# |
|  | PE(16:0/18:3(9Z,12Z,15Z)) | 4098443±602452 | 2177365±313959** | 2095491±544347 | 2283741±285033 | 2630202±336430## |
|  | PE(16:0/22:5(7Z,10Z,13Z,16Z,19Z)) | 4330557±648946 | 3626472±469890** | 4234136±649293# | 4152939±342026# | 4497746±402122## |
| LysoPC | LysoPC(18:4(6Z,9Z,12Z,15Z)) | 125529±48631 | 311721±70595** | 165403±60254## | 114921±46958## | 175904±49533## |
|  | LysoPC(18:3(6Z,9Z,12Z)) | 2199082±409111 | 13282869±2961547** | 4709917±2350711## | 4393340±1192049## | 4639926±2305479## |
|  | LysoPC(20:4(8Z,11Z,14Z,17Z)) | 16091059±2449574 | 22239937±3625765** | 9623158±2865232## | 9517590±1423438## | 10017235±4006444## |
|  | LysoPC(22:6(4Z,7Z,10Z,13Z,16Z,19Z)) | 13544748±1408342 | 18653483±1546080** | 14943063±2350328## | 15178046±1457185## | 14503051±3085525## |
|  | LysoPC(20:4(5Z,8Z,11Z,14Z)) | 15763375±998101 | 23416815±1947767** | 19978072±1209674## | 20671472±2934352# | 20921538±2251806# |
|  | LysoPC(22:5(4Z,7Z,10Z,13Z,16Z)) | 80706870±7581966 | 139245679±15130529** | 82740052±23746722## | 84365915±10277993## | 83102606±24218274## |
|  | LysoPC(22:5(7Z,10Z,13Z,16Z,19Z)) | 433445±132454 | 3382452±509335** | 1598911±277235## | 1624718±530328## | 1793792±308553## |
|  | LysoPC(20:2(11Z,14Z)) | 6410877±1386236 | 30216257±4255594** | 13975090±3127014## | 13974059±4322425## | 15340691±2303981## |
|  | LysoPC(22:4(7Z,10Z,13Z,16Z)) | 521817±194275 | 692282±81698** | 423281±63797## | 401812±83435## | 391953±54395## |
|  | LysoPC(20:1(11Z)) | 67055368±7396274 | 78877303±11497382** | 61044004±13167747## | 59517395±10459622## | 57644966±9811852## |
|  | LysoPC(20:0/0:0) | 10928443±2098349 | 14528576±2247953** | 11070829±2263670## | 15892025±2157198 | 13548494±1831001 |
|  | LysoPC(16:0) | 261863±33226 | 184254±21518** | 245207±43112## | 255107±25802## | 256098±39174## |
| PI | PI(18:0/18:2(9Z,12Z)) | 12072479±2999243 | 8816622±1620197** | 11636655±2246173## | 10726281±3528932 | 11278558±2483324# |
|  | PIP2(16:0/16:1(9Z)) | 1741±1005 | 20374406±5693424** | 24113567±12837637 | 26026882±10998609 | 22524416±8659072 |
| Cer | Cer(d18:1/24:1(15Z)) | 300355±110710 | 146607±96529** | 302365±158429# | 505885±359136## | 349649±218927# |
| Other metabolites | 8,11,14-Eicosatrienoic acid | 86825440±7759325 | 100973473±10778593** | 80009997±21747112# | 73903762±11771825## | 66295907±10545640## |
|  | Deoxycholic acid | 757284±505914 | 19221098±5530500** | 14351641±2737261# | 11632439±3975607## | 7844598±3517783## |
|  | Glycocholic acid | 29672298±15371408 | 10727414±6904285** | 20169872±10998395# | 33044549±19256241## | 21975592±7174452## |
|  | PS(18:0/18:1(9Z)) | 1488608±455046 | 1066344±102741* | 2113608±1011576## | 2579760±538041## | 2636943±610031## |
|  | Bilirubin glucuronide | 121171±64960 | 45583±13981** | 257102±142969## | 254897±288840# | 292827±272861## |
|  | Taurocholic acid | 85211351±66419043 | 2434915±2096713** | 15008732±7784126## | 19774744±17935306## | 10722241±10057235# |
|  | Alpha-Linolenic acid | 4149788±1202750 | 2963652±1154728* | 4304422±1234223# | 3402804±798407 | 3117761±474753 |
|  | 12,13-DHOME | 863492±436466 | 338326±103741** | 639401±201272## | 476985±166822# | 450961±119045# |
|  | Lithocholic acid glycine conjugate | 335851±210588 | 63518±21086** | 121409±54859## | 115916±56421# | 97766±70471 |
|  | 24-Methylenecholesterol | 260645±45152 | 130342±18411** | 167400±39987# | 167895±50701# | 162956±41834# |
|  | 5b-Cyprinol sulfate | 2142958±1519217 | 824677±222309** | 1199234±438910# | 1297459±325879## | 1127972±365438# |
|  | D-Urobilinogen | 211336±162443 | 1862775±856990** | 715605±428161## | 971691±447033## | 984323±502498# |
|  | 25-Hydroxycholesterol | 109311±38656 | 257712±79391** | 194898±38220# | 177541±19969## | 189564±31760# |
|  | Bilirubin | 5130244±2589226 | 9520195±3415816** | 4202711±2836181## | 5385162±2633616## | 8736014±3454721 |
|  | Sphingosine | 14995935±2916407 | 22273501±3775086** | 17760873±3983692# | 18853582±2626018# | 17797441±4372454# |

**Supplementary table 6: Fragments information of fecal lipid metabolites**

| **No.** | **Compound Name** | **m/z** | **RT**  **(min)** | **Adducts** | **Formula** | **Error (ppm)** | **Mass fragments** |
| --- | --- | --- | --- | --- | --- | --- | --- |
| 1 | 17alpha,20alpha-Dihydroxypregn-4-en-3-one | 377.2323 | 6.80 | M+FA-H | C21H32O3 | -3.10 | 377.2333 [C21H32O3+FA-H], 331.2279 [C20H30O+FA-H], 313.1809 [C19H25O+FA-H], 297.1860 [C19H25+FA-H], 259.1704 [C16H22+FA-H] |
| 2 | Mesobilirubinogen | 593.3321 | 7.50 | M+H | C33H44N4O6 | -2.14 | 593.3334 [C33H44N4O6+H], 331.2279 [C20H30O+FA-H], 297.1860 [C19H25+FA-H], 271.1704 [C17H23+FA-H], 259.1704 [C16H22+FA-H] |
| 3 | 24R,25-Dihydroxyvitamin D3 | 417.3355 | 7.62 | M+H | C27H44O3 | -1.86 | 417.3363 [C27H44O3+H], 381.3152 [C27H41O-e], 253.1951 [C19H25-e], 171.1169 [C13H16-e], 145.1012 [C11H12+H] |
| 4 | Dehydroepiandrosterone | 289.2154 | 7.82 | M+H | C19H28O2 | -2.83 | 289.2162 [C19H28O2+H], 229.1951 [C17H24+H], 175.1482 [C13H18+H], 161.1325 [C12H16+H], 131.0856 [C10H11-e] |
| 5 | 11,12-DiHETrE | 337.2376 | 8.03 | M-H | C20H34O4 | -2.47 | 337.2384 [C20H34O4-H], 319.2278 [C20H32O3-H], 301.2173 [C20H31O2-H], 291.2329 [C19H32O2-H], 209.1547 [C13H23O2-H] |
| 6 | Sphinganine | 302.3046 | 8.07 | M+H | C18H39NO2 | -2.60 | 302.3054 [C18H39NO2+H], 285.3026 [C18H38NO+H], 283.2870 [C18H37NO-e], 266.2843 [C18H36N-e], 254.2843 [C17H34N+H] |
| 7 | 3a,7a,12a-Trihydroxy-5b-cholestan-26-al | 433.3311 | 8.33 | M-H | C27H46O4 | -2.93 | 433.3323 [C27H46O4-H], 415.3217 [C27H44O3-H], 389.3061 [C25H42O3-H], 371.2955 [C25H40O2-H], 333.2435 [C21H35O3-H] |
| 8 | LysoPC(18:1(9Z)) | 522.3542 | 8.69 | M+H | C26H52NO7P | -2.33 | 522.3554 [C26H52NO7P+H], 339.2894 [C21H39O3-e], 258.1101 [C8H19NO6P+H], 104.1070 [C5H13NO+H], 86.0965 [C5H13N-e] |
| 9 | PE(15:0/20:0) | 734.5724 | 8.77 | M+H | C40H80NO8P | 4.11 | 717.5429 [C40H78O8P-e], 699.5324 [C40H76O7P-e], 57.0699 [C4H9-e] |
| 10 | LysoPC(17:0) | 554.3446 | 8.81 | M+FA-H | C25H52NO7P | -3.37 | 554.3463 [C25H52NO7P+FA-H], 494.3252 [C24H49NO7P+e], 283.2642 [C18H35O2+e], 269.2486 [C17H33O2+e], 255.2330 [C15H31+FA-H] |
| 11 | LysoPC(18:0) | 568.3604 | 8.89 | M+FA-H | C26H54NO7P | -2.99 | 568.3620 [C26H54NO7P+FA-H], 508.3408 [C25H51NO7P+e], 283.2642 [C18H35O2+e], 255.2330 [C15H31+FA-H], 242.0799 [C6H15NO4P+FA-H] |
| 12 | LysoPC(20:0/0:0) | 552.4020 | 9.47 | M+H | C28H58NO7P | -0.74 | 552.4024 [C28H58NO7P+H], 534.3919 [C28H57NO6P-e], 184.0733 [C5H13NO4P+H], 104.1070 [C5H13NO+H], 85.1012 [C6H13-e] |
| 13 | 4,4-Dimethyl-5a-cholesta-8,24-dien-3-b-ol | 457.3672 | 9.47 | M+FA-H | C29H48O | -3.72 | 456.3609 [C29H48O+FA-H], 413.3425 [C27H44+FA-H], 397.3112 [C26H40+FA-H], 395.2956 [C26H39+FA-H] |
| 14 | 24-Hydroxycholesterol | 403.3563 | 9.58 | M+H | C27H46O2 | -1.84 | 403.3571 [C27H46O2+H], 385.3465 [C27H45O-e], 367.3360 [C27H43-e], 215.1795 [C16H22+H], 173.1325 [C13H18-e] |
| 15 | 3 beta-Hydroxy-5-cholestenoate | 415.3207 | 10.10 | 2M-H | C27H44O3 | -2.62 | 415.3217 [C27H44O3-H], 397.3112 [C27H42O2-H], 371.3319 [C26H43O+e], 353.2850 [C25H38O-H], 205.1598 [C14H22O-H] |
| 16 | 22b-Hydroxycholesterol | 403.3560 | 10.12 | M+H | C27H46O2 | -2.68 | 403.3571 [C27H46O2+H], 215.1795 [C16H22+H], 177.1274 [C12H18O-e], 161.1325 [C12H16+H], 147.1169 [C11H14+H] |
| 17 | Bilirubin | 585.2694 | 10.25 | M+H | C33H36N4O6 | -2.27 | 585.2708 [C33H36N4O6+H], 299.1391 [C17H19N2O3-e], 284.1156 [C16H17N2O3-e], 257.1285 [C15H15N2O2+H] |
| 18 | PE(22:5(4Z,7Z,10Z,13Z,16Z)/24:1(15Z)) | 920.6387 | 10.85 | M+FA-H | C51H90NO8P | 0.11 | 920.6386 [C51H90NO8P+FA-H], 411.3480 [C24H45O2+FA-H], 309.2799 [C19H37+FA-H], 183.0064 [C3H6O4P+FA-H], 101.0608 [C4H9+FA-H] |
| 19 | SM(d18:0/16:1(9Z)) | 703.5725 | 11.28 | M+H | C39H79N2O6P | -3.32 | 703.5749 [C39H79N2O6P+H], 685.5643 [C39H78N2O5P-e], 239.2370 [C16H29O+H], 184.0733 [C5H13NO4P+H], 166.0628 [C5H13NO3P-e] |
| 20 | PC(18:1(11Z)/18:3(6Z,9Z,12Z)) | 826.5587 | 11.39 | M+FA-H | C44H80NO8P | -2.09 | 826.5604 [C44H80NO8P+FA-H], 279.2330 [C17H29+FA-H], 277.2173 [C18H29O2+e], 255.2330 [C15H29+FA-H] |
| 21 | PC(16:0/22:6(4Z,7Z,10Z,13Z,16Z,19Z)) | 850.5584 | 11.59 | M+FA-H | C46H80NO8P | -2.45 | 850.5604 [C46H80NO8P+FA-H], 790.5392 [C45H77NO8P+e], 327.2329 [C22H31O2+e], 283.2431 [C21H31+e], 255.2329 [C16H31O2+e] |
| 22 | Cer(d18:1/24:1(15Z)) | 692.6181 | 11.75 | M+FA-H | C42H81NO3 | -2.67 | 692.6198 [C42H81NO3+FA-H], 646.6143 [C42H81NO3+e], 390.3741 [C26H48NO+e], 255.2330 [C15H29+FA-H], 241.2173 [C14H28+FA-H] |
| 23 | PC(18:2(9Z,12Z)/16:0) | 802.5584 | 11.79 | M+FA-H | C42H80NO8P | -2.55 | 801.5525 [C42H80NO8P+FA-H], 724.5392 [C41H77NO8P+e], 279.2329 [C18H31O2+e], 255.2329 [C16H31O2+e] |
| 24 | PC(18:1(11Z)/20:4(5Z,8Z,11Z,14Z)) | 852.5751 | 11.85 | M+FA-H | C46H82NO8P | -1.12 | 852.5760 [C46H82NO8P+FA-H], 303.2329 [C20H31O2+e], 281.2486 [C18H33O2+e] |
| 25 | 7alpha-Hydroxy-3-oxo-4-cholestenoate | 429.3003 | 11.86 | M-H | C27H42O4 | -1.71 | 429.3010 [C27H42O4-H], 403.3217 [C26H42O3+e], 385.3112 [C26H41O2+e], 367.3006 [C26H39O+e], 109.0658 [C7H8O+e] |
| 26 | Ceramide (d18:1/9Z-18:1) | 608.5245 | 11.91 | M+FA-H | C36H69NO3 | -4.82 | 608.5259 [C36H69NO3+FA-H], 562.5204 [C36H69NO3+e], 532.5099 [C35H66NO2+e], 306.2802 [C20H36NO+e], 280.2645 [C18H34NO+e] |
| 27 | 7-Dehydrodesmosterol | 383.3297 | 11.93 | M+H | C27H42O | -2.97 | 383.3309[C27H42O+H], 325.2890 [C24H36+H], 253.1951 [C19H25-e], 239.1795 [C18H24-e], 171.1169 [C13H16-e] |
| 28 | Docosahexaenoic acid | 655.4733 | 11.97 | 2M-H | C22H32O2 | 0.17 | 327.2329 [C22H32O2-H], 283.2431 [C21H31+e], 229.1961 [C17H25+e], 59.0138 [C2H3O2+e] |
| 29 | PC(14:0/22:2(13Z,16Z)) | 808.5825 | 12.07 | M+Na | C44H84NO8P | -0.18 | 808.5827 [C44H84NO8P+Na], 807.5748 [C44H84NO8P+Na], 357.2764 [C22N39O2+Na], 175.1457 [C11H19+Na], 161.1301 [C10H17+Na] |
| 30 | PC(18:0/20:4(5Z,8Z,11Z,14Z)) | 854.5902 | 12.63 | M+FA-H | C46H84NO8P | -1.76 | 854.5917 [C46H84NO8P+FA-H], 303.2329 [C20H31O2+e], 283.2642 [C18H35O2+e], |
| 31 | PC(16:0/20:2(11Z,14Z)) | 830.5903 | 12.65 | M+FA-H | C44H84NO8P | -1.76 | 830.5917 [C44H84NO8P+FA-H], 770.5705 [C43H81NO8P+e], 307.2642 [C20H35O2+e], 281.2486 [C17H31+FA-H], 255.2329 [C16H31O2+e] |
| 32 | Cholesterol | 387.3612 | 12.80 | M+H | C27H46O | -2.56 | 409.3441 [C27H46O+NA], 369.3516 [C27H45-e], 339.2658 [C22H35O+Na], 243.2108 [C18H26+H], 215.1795 [C16H22-e] |
| 33 | SM(d18:1/20:0) | 803.6280 | 13.19 | M+FA-H | C43H87N2O6P | -0.55 | 803.6284 [C43H87N2O6P+FA-H], 743.6072 [C42H84N2O6P+e], 255.2330 [C15H29+FA-H], 59.0139 [CH3+FA-H] |
| 34 | Alpha-Linolenic acid | 555.4426 | 13.24 | 2M-H | C18H30O2 | 1.27 | 555.4419 [C18H30O2+e+M], 537.4313 [C18H28O-H+M], 511.4520 [C17H29+e+M], 69.0345 [C4H5O+e] |
| 35 | PE(14:1(9Z)/16:1(9Z)) | 660.4613 | 13.52 | M+H | C35H66NO8P | 2.13 | 643.4334 [C35H64O8P-e], 97.1012 [C7H13-e], 85.1012 [C6H13-e], 83.0856 [C6H11-e], 71.0856 [C5H11-e] |
| 36 | Ceramide (d18:1/12:0) | 610.5400 | 13.67 | M+FA-H | C36H71NO3 | -2.77 | 610.5416 [C36H71NO3+FA-H], 534.5255 [C35H86NO2+e] |
| 37 | Ceramide (d18:1/20:0) | 638.5715 | 13.86 | M+FA-H | C38H75NO3 | -2.40 | 638.5729 [C38H75NO3+FA-H], 592.5674 [C38H75NO3+e], 562.5568 [C37H72NO2+e], 392.3534 [C25H46NO2+e], 336.3271 [C22H42NO+e] |

**Supplementary table 7: Intensity of fecal metabolites**

| **Metabolite type** | **Compound Name** | **NC/ Intensity** | **HFD/ Intensity** | **XZP-L/ Intensity** | **XZP-M/ Intensity** | **XZP-H/ Intensity** |
| --- | --- | --- | --- | --- | --- | --- |
| PC | PC(18:2(9Z,12Z)/16:0) | 6689604±1267473 | 876884±235049** | 2112639±697639## | 1880201±819685## | 1180881±294512# |
|  | PC(18:1(11Z)/20:4(5Z,8Z,11Z,14Z)) | 192826±52702 | 6994±1544** | 37882±28062## | 17203±7593## | 9030±6280 |
|  | PC(18:1(11Z)/18:3(6Z,9Z,12Z)) | 1370776±326512 | 204350±85418** | 510674±233652## | 713122±484095## | 393472±184572## |
|  | PC(18:0/20:4(5Z,8Z,11Z,14Z)) | 176707±107735 | 54708±32091** | 144492±73059## | 113223±47092## | 74356±33924 |
|  | PC(16:0/22:6(4Z,7Z,10Z,13Z,16Z,19Z)) | 460474±80928 | 126574±42594** | 247841±51966 ## | 189989±64290 # | 156679±55084 |
|  | PC(16:0/20:2(11Z,14Z)) | 1701753±383883 | 321795±108305** | 729193±168780## | 750562±90645## | 440447±114699# |
|  | PC(14:0/22:2(13Z,16Z)) | 424919±127440 | 95226±38602** | 449483±146645## | 403491±175010## | 220042±85829## |
| SM | SM(d18:0/16:1(9Z)) | 8130479±1167232 | 84746111±21285457** | 51430690±18577990## | 62895856±22007373# | 61306802±14050915## |
|  | SM(d18:1/20:0) | 169030±75146 | 545999±126185** | 256433±125280## | 355151±131533## | 239691±93558## |
| PE | PE(22:5(4Z,7Z,10Z,13Z,16Z)/24:1(15Z)) | 117472±21677 | 2645873±645834** | 1741610±400544## | 1554246±292171## | 2820106±981331 |
|  | PE(15:0/20:0) | 548117±242748 | 907372±303870** | 576958±301870# | 569602±388213# | 650999±205648# |
|  | PE(14:1(9Z)/16:1(9Z)) | 6886±3791 | 476282±159666** | 332834±135749# | 345083±78067# | 355464±58915# |
| Cer | Cer(d18:1/24:1(15Z)) | 58597±32792 | 260959±127945** | 136995±115423# | 50275±11001## | 244150±100543 |
|  | Ceramide (d18:1/20:0) | 406003±121480 | 669578±130490** | 672705±235798 | 654233±244858 | 385307±138653## |
|  | Ceramide (d18:1/12:0) | 618315±278019 | 1453339±402221** | 950933±324255## | 1051272±373276# | 1055849±260888# |
|  | Ceramide (d18:1/9Z-18:1) | 6398474±846808 | 13429672±2051685** | 10467860±3748166## | 9485720±3278978## | 11541640±3442645 |
| LysoPC | LysoPC(17:0) | 81543±29455 | 574671±271254** | 316569±131865# | 423469±286304 | 508293±271130 |
|  | LysoPC(20:0/0:0) | 303298±42486 | 1670424±638898** | 1013591±515179# | 1021709±338950# | 1108910±439557# |
|  | LysoPC(18:0) | 128985±24401 | 1014207±556519** | 548779±307015# | 949554±705028 | 874552±479218 |
|  | LysoPC(18:1(9Z)) | 38976464±11512606 | 81717272±25022820** | 54209095±18486288# | 58692766±14283640# | 81841876±21437496 |
| Other metabolites | Sphinganine | 19076941±6269675 | 12408230±4347832* | 25178268±10011868## | 10303442±5927477 | 12649990±3662018 |
|  | Cholesterol | 4683239±357188 | 23813813±8359318** | 31070710±6100410# | 22595579±5176010 | 26618977±4877325 |
|  | Dehydroepiandrosterone | 287006±37700 | 861198±146463** | 1011247±139701## | 1220471±409358## | 923488±44630## |
|  | 3a,7a,12a-Trihydroxy-5b-cholestan-26-al | 626189±97023 | 1293881±342861** | 1130127±213429 | 925891±275364# | 1026100±177811# |
|  | 17alpha,20alpha-Dihydroxypregn-4-en-3-one | 77817±20298 | 395537±50469** | 292924±136393# | 322746±74974# | 445655±133309 |
|  | 7-Dehydrodesmosterol | 24732387±1297925 | 165708702±8490565** | 138745478±38804794# | 151256997±8395128## | 149756398±11903181## |
|  | 4,4-Dimethyl-5a-cholesta-8,24-dien-3-b-ol | 221733±73796 | 326769±86704** | 179913±60571## | 177197±42776## | 252367±65971# |
|  | 22b-Hydroxycholesterol | 3508792±405710 | 15252170±1782995** | 13523076±1695865# | 12678218±2541903# | 12327550±1754255## |
|  | Alpha-Linolenic acid | 23034±9930 | 3294±1147** | 5055±2097# | 6235±2491## | 3990±1723 |
|  | Docosahexaenoic acid | 383±216 | 4407±2300** | 3024±1472 | 1479±683## | 2286±1347# |
|  | 24R,25-Dihydroxyvitamin D3 | 6263049±712631 | 8702405±954199** | 7039633±1227495## | 7104714±1092018## | 7062707±665144## |
|  | 24-Hydroxycholesterol | 753710±93586 | 2140785±377680** | 1784418±226877# | 1652597±366717## | 1730294±189029## |
|  | 11,12-DiHETrE | 212217±28301 | 552453±229860** | 367205±105864# | 368710±148553# | 386934±95549# |
|  | 3 beta-Hydroxy-5-cholestenoate | 297426±154494 | 10993±9981** | 29913±17419## | 10631±9452 | 27825±23275# |
|  | 7alpha-Hydroxy-3-oxo-4-cholestenoate | 2271±1146 | 3835±922** | 2681±774## | 2162±921## | 2982±727# |
|  | Mesobilirubinogen | 6698166±2964058 | 51696198±16496038** | 33010533±13947425# | 37171187±13377467# | 27907170±14271533## |
|  | Bilirubin | 3397961±1488107 | 2161766±907240** | 3155010±1056273# | 4511078±2813381# | 3342177±1332278# |

**Supplementary table 8: Pathway analysis of serum, liver and feces biomarkers with MetPA**

| **NO.** | **Pathway** | **Total** | **Expected** | **Hits** | **Raw p** | **Impact** |
| --- | --- | --- | --- | --- | --- | --- |
| 1 | Primary bile acid biosynthesis | 46 | 1.1574 | 10 | 0.0000 | 0.1358 |
| 2 | Sphingolipid metabolism | 21 | 0.5284 | 6 | 0.0000 | 0.5456 |
| 3 | Arachidonic acid metabolism | 36 | 0.9058 | 5 | 0.0017 | 0.0000 |
| 4 | Steroid biosynthesis | 42 | 1.0568 | 5 | 0.0033 | 0.1149 |
| 5 | Linoleic acid metabolism | 5 | 0.1258 | 2 | 0.0059 | 1.0000 |
| 6 | Glycerophospholipid metabolism | 36 | 0.9058 | 4 | 0.0113 | 0.2633 |
| 7 | Biosynthesis of unsaturated fatty acids | 36 | 0.9058 | 4 | 0.0113 | 0.0000 |
| 8 | alpha-Linolenic acid metabolism | 13 | 0.3271 | 2 | 0.0404 | 0.3333 |
| 9 | Steroid hormone biosynthesis | 85 | 2.1387 | 5 | 0.0582 | 0.0639 |
| 10 | Porphyrin and chlorophyll metabolism | 30 | 0.7548 | 2 | 0.1730 | 0.0529 |
| 11 | Taurine and hypotaurine metabolism | 8 | 0.2013 | 1 | 0.1848 | 0.0000 |
| 12 | Glycosylphosphatidylinositol (GPI)-anchor biosynthesis | 14 | 0.3523 | 1 | 0.3011 | 0.0040 |
| 13 | Pentose and glucuronate interconversions | 18 | 0.4529 | 1 | 0.3695 | 0.1406 |

# Data Availability Statement

The datasets presented in this study can be found in online at:

https://www.jianguoyun.com/p/Daq-2x8Q_7WqCxjwl_QEIAA

The name of the repository and accession number can be found below: NCBI - PRJNA928847
